# Me‐4PACz Functionalized MXene for Halide Perovskite Solar Cells

**DOI:** 10.1002/advs.202509898

**Published:** 2025-08-14

**Authors:** Masoud Karimipour, Nil Monrós Oliveras, Zhenchuan Tian, Francesco Salutari, Maria Chiara Spadaro, Tiankai Zhang, Naji Vahedigharehchopogh, Jordi Arbiol, Feng Gao, Monica Lira‐Cantu

**Affiliations:** ^1^ Catalan Institute of Nanoscience and Nanotechnology (ICN2) CSIC and BIST Campus UAB Bellaterra Barcelona Catalonia 08193 Spain; ^2^ Department of Physics and Astronomy “Ettore Majorana” University of Catania and CNR‐IMM Catania 95123 Italy; ^3^ Department of Physics, Chemistry and Biology. (IFM) Linköping University Linköping 58183 Sweden; ^4^ ICREA, Pg. Lluís Companys 23 Barcelona Catalonia 08010 Spain

**Keywords:** impedance spectroscopy, interfacial passivation, MXene, operational stability, perovskite solar cells, trap density

## Abstract

Interfacial passivation employing 2D Ti_3_C_2_ MXenes has proved to be an excellent strategy to optimize band alignment and passivate defects, leading to the reduction of non‐radiative recombination in Perovskite Solar Cells (PSCs). Here in, the synthesis and functionalization of Ti_3_C_2_ MXene are reported with the [4‐(3,6‐Dimethyl‐9*H*‐carbazol‐9‐yl)butyl]phosphonic acid molecule (MXene:Me‐4PACz), which is proved by XRD and HRTEM‐EELS analyses. Its application at the interface between the halide perovskite (HP) and the Spiro‐OMeTAD in normal configuration PSCs, results in the enhancement of indoor and outdoor stability. The MXene:Me‐4PACz nanomaterial is obtained in the form of nanoneedles, which, applied in complete PSCs, resulted in a power conversion efficiency (PCE) of ≈21.5%, in comparison with the control device with ≈20.1%. The modified device showed a T_88_ operational stability obtained at 1000 h for ISOS‐L‐1 and T_50_ at ≈1000 h for ISOS‐O‐2. While, all the control devices degraded 55% after 1000 h under ISOS‐L and almost 100% after 900 h under ISOS‐O‐2. Characterization analyses indicate that the efficiency and stability enhancement is due to the improved energy band alignment and charge extraction, to the increased perovskite surface hydrophobicity, and the significant reduction of deep and shallow trap states.

## Graphical abstract

Functionalized Ti₃C₂ MXene nanoneedles with Me‐4PACz employed at the interface between perovskite and Spiro‐OMeTAD, improve charge extraction, energy alignment, and surface hydrophobicity. Devices show boosted PCE (21.5%) and operational stability (T88 = 1000 h, ISOS‐L‐1), highlighting MXene:Me‐4PACz as a powerful strategy for simultaneously improving efficiency and outdoor durability of perovskite solar cells.

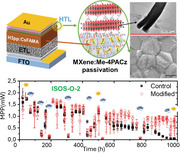

## 1 Introduction

Halide perovskite solar cells have attracted the interest of industry due to the excellent performance the technology has demonstrated at the academic level. The commercialization of the technology appears imminent, and the crucial key for deployment is their operational stability under real outdoor conditions.^[^ ^1^ ^]^ Many studies have been dedicated to the study of the operational stability of perovskite solar cells by applying different engineering techniques such as compositional engineering,^[^ ^2^ ^]^ additive engineering to stabilize phase transformation of perovskite,^[^ ^3^ ^]^ trap density passivation engineering,^[^ ^4^ ^]^ interfacial engineering,^[^ ^5^ ^]^ band alignment engineering,^[^ ^6^ ^]^ configurational engineering,^[^ ^7^ ^]^ among others. The key factor in most of the studies is the control of the perovskite degradation by means of immobilizing ions in the bulk of the perovskite or at the interfaces.^[^ ^8^, ^9^ ^]^ Some of these techniques have been employed to increase the operational stability of perovskite solar cells such as interfacial engineering using organic molecules,^[^ ^10^ ^]^ 2D/3D passivation,^[^ ^11^, ^12^ ^]^ inorganic 2D materials,^[^ ^13^ ^]^ additive engineering,^[^ ^14^ ^]^ buried interface passivation,^[^ ^15^ ^]^ enhancement of band alignment,^[^ ^16^ ^]^ etc.

Tan et al^[^ ^11^ ^]^ harnessed organic ammonium iodide, phenyl trimethylammonium iodide (PTAI), to form the 1D (PTAPbI_3_) and 2D (PTA_2_PbI_4_) perovskite distribute at the grain boundaries and the film surface of CsPbI_3_, the champion device recorded a 21.0% of PCE, 1.2 V of Voc, 20.86 mA cm^−2^ of Jsc and 0.84 of fill factor, as well as good stability with 98% of initial PCE over 500 h under continuous light illumination with N_2_ atmosphere. Wu et al.^[^ ^12^ ^]^ used PEMA, 2‐(4‐amino phenyl) ethylamine cation, to construct a Dion–Jacobson 2D phase that covers the surface of the 3D CsPbI_3_ phase, which achieved 19.5% of PCE with Voc of 1.197 V, Jsc of 20.06 mA cm^−2^, and 0.81 of FF, after 1260 h of maximum power point tracking with 1.2 sun illumination, it still maintains good stability with 83% of PCE. Bi et al.^[^ ^10^ ^]^ synthesized a bifunctional molecular modulator, 3‐(5‐mercapto‐1H‐tetrazol‐1‐yl) benzenaminium iodide (SN), to induce the formation of large‐grain crystals with high quality and passivate the perovskite surface defects. Consequently, the device achieved over 20% PCE with a larger area (> 1 cm^2^) and showed good stability. After exceeding 500 h of operation under full sun intensity in a 20% RH atmosphere, the device still maintained ≈20% PCE. Cao et al^[^ ^13^ ^]^ introduced WS_2_ interlayer as a growth template for mixed (CsMAFA) perovskite to form WS_2_/perovskite heterojunction along (001) direction. Ultimately, the champion device shows PCE up to 21.1%, and 1.15 V of Voc, 22.75 mA cm^−2^ of Jsc, and 0.806 of FF. The non‐encapsulated device exposed to ambient air with ≈30% humidity for one month still retains 90% of its initial PCE. Many studies have been performed to investigate the chemical effect of different functional groups on passivation of lead halide perovskites and their interfacial contacts with neighboring layers,^[^ ^14^, ^17^, ^18^, ^19^ ^]^ such as amine groups,^[^ ^14^ ^]^ which are famous for open circuit enhancement of PSCs, carboxylic groups for enhancement of attachment to ETL/HTL layers and charge extraction.^[^ ^17^ ^]^ Recently, phosphonic acid groups were introduced to passivate the shallow trap states and enhance the stability of the PSCs while preserving the device performance parameters.^[^ ^18^ ^]^ In addition, Zhang et al.^[^ ^19^ ^]^ revealed that MPA‐CPA, (2‐(4‐(bis(4‐methoxyphenyl)amino)phenyl)‐1‐cyanovinyl)phosphonic acid passivation can mitigate buried interfacial defects and enhance the stability of the PSCs significantly. They have reported that the cyano and phosphonic groups can synergistically coordinate with lead ions to passivate the interfaces and increase both the PCE up to 25.2% and stability with retaining > 90% of its initial PCE after 2000 h operation under ISOS‐L‐1. For example, Xie et al. have used H3pp in the bulk of 4‐cation lead halide perovskite for the passivation of shallow traps and extending the device stability.^[^ ^18^ ^]^ Another interesting type of phosphonic acid materials is PACz molecules, which have shown hole‐extracting properties and have been used extensively for the fabrication of high efficiency inverted perovskite solar cells,^[^ ^20^, ^21^, ^22^ ^]^ such as Me‐4PACz,^[^ ^20^ ^]^ 2PACz,^[^ ^20^ ^]^ MeO‐4PACz,^[^ ^21^ ^]^ MeO‐2PACz.^[^ ^22^ ^]^ The main idea is to use a thin layer of several nanometer in thickness, of such organic molecules directly on the transparent conductive oxide (TCO) substrates as HTL. For example, Chen et al.^[^ ^23^ ^]^ have reported the highest inverted efficiency of 26.15% competitive to the normal configuration. In another report, Kim et al.^[^ ^24^ ^]^ employed a combination of Me‐4PACz and MeO‐2PACz to fabricate high efficiency PSCs reaching PCE of 21.09%, FF of 0.79, Voc of 1.13, and Jsc of 23.51 mA cm^−2^. The use of a mixture of the two molecules resides in the higher charge extraction abilities of MeO‐2PACz and the good adhesion to the surface ability of Me‐4PACz. MeO‐2PACz induce hydrophobicity on the surface of ITO, and therefore, perovskite deposition results in a nonhomogeneous and non‐uniform thin film. The introduction of Me‐4PACz mitigates this problem.

On the other hand, MXene nanosheets have found their pass way through advanced scientific areas of technology from large scale flexible electronics,^[^ ^25^ ^]^ biosensors,^[^ ^26^ ^]^ batteries,^[^ ^27^ ^]^ and capacitors^[^ ^28^ ^]^ up to solar cell revelation.^[^ ^15^, ^16^, ^29^, ^30^, ^31^, ^32^ ^]^ Gogotsi et al. most recently have proved that large thin films of Ti_3_C_2_ MXene nanoflakes can be stable at ambient atmosphere for several years, and it has proved the prospective stability of these nanosheets for futuristic applications.^[^ ^33^ ^]^ Agresti et al.^[^ ^29^ ^]^ has introduced MXene with various terminal groups into both perovskite absorber, TiO_2_ layer, and the interlayer between the perovskite and TiO_2_ ETL. The device exhibited PCE over 20%. Similarly, Guo et al.^[^ ^30^ ^]^ utilized MXene nanosheet with halogen‐terminal (T_X_ = F, Cl, Br, I) as an additive to connect perovskite through interfacial ionic‐bonding for inhibiting the lattice instability of FAPbI_3_ perovskite. As a result, the strong ionic interaction of Pb‐T_x_ could reduce the lattice instability and improve device stability. He et al.^[^ ^31^ ^]^ have incorporated Ti_3_C_2_Cl_X_ nano‐MXene into the ETL/PVK interface, the formation of Pb–Cl can boost carrier extraction and hinder the non‐radiative recombination. Likewise, a kind of fluorine‐rich, oxygen‐containing, and hydroxy‐free Ti_3_C_2_T_X_ MXene nanosheets was synthesized by Zhao et al^[^ ^32^ ^]^ to serve as an additive within the perovskite layer, the MXene distributed at the grain boundary of perovskite crystalline can enhance carrier transport, meanwhile, the fluorine groups possess the capability to form hydrogen bond with FA^+^, and coordinate to Pb^2+^, which could restrain the ion immigration and accumulation, as well as passivate the defects along the grain boundary at the interface. Most recently, Cao et al. have reported buried interfacial passivation of n‐i‐p configurational PSCs using Cl‐Ti_3_C_2_ for both passivation of nonradiative recombination at ETL (SnO_2_ nanoparticle layer)/perovskite interface and work function alignment to enhance both PCE up to 25.09% and with substantial stability enhancement. They reported the modified device retains more than 90% of MPP value after > 350 h under continuous LED (100 W cm^−2^) illumination, N_2_ atmosphere, and at 45–55 °C.^[^ ^15^ ^]^

Recently, we reported the use of MXene nanosheets functionalized with the H3pp molecule employed to passivate the interfacial defects between halide perovskite (HP) layer and Spiro‐OMeTAD hole transport layer (HTL). We selected the H3pp additive to passivate both HP layer and the MXene nanosheet layer to create a continuous connection between the passivated HP layer and MXene nanosheets to overcome the band alignment mismatch and hinder charge recombination at the interface of HP/HTL.^[^ ^16^ ^]^ Our work proved that this type of passivation increases the hydrophobicity of the perovskite surface and lowers defect density, enhancing outdoor operational stability up to 600 h.

In this work, we applied [4‐(3,6‐Dimethyl‐9H‐carbazol‐9‐yl) butyl] phosphonic acid (Me‐4PACz), an organic molecule exhibiting good charge extraction properties, for the functionalization of MXenes. The as‐prepared MXene:Me‐4PACz thin film was deposited on the surface of the halide perovskite showing a nanoneedle‐like morphology. The MXene:Me‐4PACz thin film was then applied as an interface between the halide perovskite and the HTL in a Perovskite Solar Cell (PSC). Our strategy resulted in the enhancement of the interfacial passivation and hole extraction properties of the PSC, improving the operational stability of the PSC under indoor and outdoor stability analysis up to 1000 h. We successfully demonstrated that the application of MXene:Me‐4PACz, enhances charge extraction properties, reduces both deep and shallow trap states, induces hydrophobicity, and extends significantly the operational stability of the devices.

## 2 Results and Discussion

MXene thin films were made via delamination and functionalization of MXenes. Briefly, the delamination was carried out through simultaneous treatment with TPAOH and hydrazine, where 10 mg of Ti_3_C_2_ Bulk MXene was mixed with 1 mL of hydrazine hydrate and 3 mL of TPAOH. The mixture was stirred at room temperature for 24h. Then, 3 mL of TBAOH (58%) and 3 mL of TMAOH were added separately and stirred overnight and for four hours, respectively, as shown in Figure S1 (Supporting Information). Once delaminated, the MXene was filtered and washed, and re‐dispersed in isopropanol (IPA) to form a green dispersion. The functionalization was then carried out by the addition of 1–2 mgs of Me‐4PACz to 2 mL of the green dispersion (delaminated Ti_3_C_2_T_X_ solution) and treated by bath ultrasonication for 20 min.

**Figure 1:**
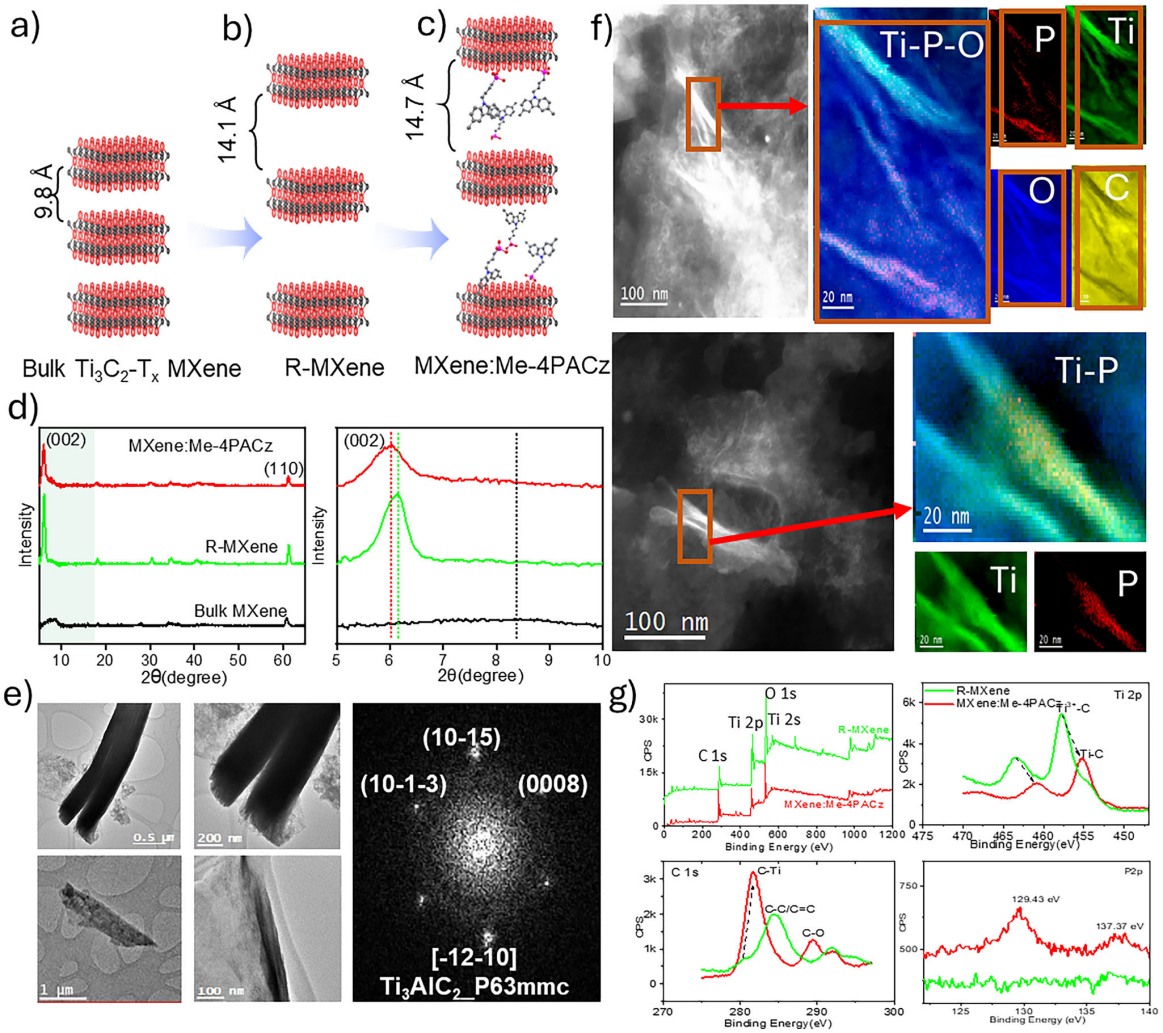
Schematical presentation of a) Bulk MXene, b) MXene nanoribbons atomic planes, c) Functionalized MXene nanoribbons with Me‐4PACz molecules, d) XRD plots of corresponding MXenes from a‐c, e) HRTEM, TEM images and corresponding power spectrum of Me‐4PACz:MXenes nanoribbons, f) STEM‐HAADF micrographs of Me‐4PACz:MXenes nanoribbons together with the corresponding STEM‐EELS compositional maps showing the different elemental distributions: C (yellow), Ti (green), P (red), and O (blue), g) XPS survey, Ti 2p, C1s, and P 2p orbital scan plots of delaminated and Me‐4PACz functionalized MXene powders.

**Figure** **1**. Shows a schematic representation of the bulk MXene (**MXene**, Figure 1a), its delamination (**R‐MXene**, Figure 1b), and its functionalization with the Me‐4PACz molecules (**MXene:Me‐4PACz**, Figure 1c). The crystalline phase identification of the different MXenes was carried out by XRD diffraction analysis. Figure 1d shows the XRD diffractograms indicating that the delamination and functionalization of MXene resulted in an increase of the interlayer distance between the MXene planes in the (002) direction. By applying Scherrer formula and Bragg diffraction law,^[^ ^34^ ^]^ the calculated number of atomic planes was between 7 and 11, as represented in Figure 1a–c (for detailed values see Table S1, Supporting Information).

A MXene:Me‐4PACz thin film was deposited on top of the halide perovskite and showed a nanoneedle‐like morphology with a thickness in the range of a few nanometers. High‐resolution transmission electron microscopy (HRTEM) analyses with the corresponding power spectrum (FFT), were made on several samples. Our results confirmed the formation of a MXene:Me‐4PACz with nanoneedle‐type structure and dimensions of ≈2–3 µm long and 200–400 nm in diameter, as shown in Figure 1e (see also Figure S2, Supporting Information). A detailed cross‐sectional HRTEM image shows the number of atomic planes observed, which lie between 6‐11, in excellent agreement with the XRD analysis (Table S1, Supporting Information). To investigate further the position of the Me‐4PACz molecules within the 2D MXene, energy electron loss spectroscopy (EELS) analyses were employed together with scanning transmission microscopy (SEM).^[^ ^35^ ^]^ Figure 1f shows the STEM‐HAADF results, including the Ti, P, and O elemental mapping of two different areas of the MXene sample (see red boxes in Figure 1f). The results show the signal of the P atoms of the phosphonate functional group of the Me‐4PACz molecule, concentrated at the edge of the MXene sample. The stronger intensity of the P signal in such areas indicates that the Me‐4PACz is successfully confined within the interlayer spacings of the 2D MXene.

To analyze further the interaction of the Me‐4PACz with the MXene and the effect on the MXene electronic structure, we carried out XPS analyses for Ti 2p, C 1s, and P 2p orbitals for the delaminated (R‐MXene) and the functionalized (MXene:Me‐4PACz) samples. Figure 1g shows the obtained Ti 2p, P 2p, and C 1s orbital scans for the powdered samples, respectively. The Ti 2p_3/2_(2p_1/2_) orbital energies for the delaminated and functionalized MXene are shown in Figure 1g. Two peaks are observed at 457.8 and 463.3 eV, which, after functionalization with the Me‐4PACs, are shifted to 455.1 and 460.9 eV, respectively. These energy values correspond to C‐Ti^3+^‐(O/OH) and C─Ti bonding type, respectively, characteristic of MXenes.^[^ ^36^, ^37^ ^]^ These shifts in MXene peaks demonstrate a significant change in the electronic energy of the MXene after functionalization. Similar results are observed for the C 1 s orbital scan, which corresponds to the carbon site binding, as shown in Figure 1g. Here we can also observe the shifting of the peaks and the formation of new ones. Especially the Ti─C bond signal at 281.7 eV is observed amplified, and the C–O binding peak is also shifted to 289.1 eV.^[^ ^36^ ^]^ We attribute these changes to the increase of the interlayer distance between MXene atomic planes (which can strengthen the in‐plane Ti‐C bonding), and to the attachment of Me‐4PCAz molecule from the ─OH functional group to the C atoms of the MXene,^[^ ^36^ ^]^ respectively. Finally, the orbital binding energies corresponding to P 2p for the P─O functional group,^[^ ^38^ ^]^ are shown in Figure 1g at 129.4 and 137.4 eV. These peaks are only observed for the functionalized MXene:Me‐4PACz sample, as an indication of the presence of Me‐4PACz molecule within the MXene structure, as expected.

The interaction between the organic molecule and the MXene is further confirmed through FTIR analysis (Figure S3, Supporting Information). The functionalized sample shows absorption bands not observed for the delaminated R‐MXene, at ≈1739 and 2900 cm^−1^, which are attributed to P–O functional group and ─CH_3_ functional group of Me‐4PACz molecules.^[^ ^39^ ^]^ The FTIR analysis also confirms the presence of the Me‐4PACz structure inside the MXene.

In summary, our results indicate the successful modification of the MXene via delamination and functionalization with the Me‐4PACz molecule. The MXene:Me‐4PACz thin film was deposited on top of the halide perovskite thin film and showed a surface with a nanoneedle shape. The intercalation of the organic molecule within the interlayer space of MXenes was effectively proved, resulting in an increase of the atomic plane distance, an increase of the in‐plane Ti─C bonding order, and its attachment from the ─OH functional group to the surface of MXene.

The selection of the best organic molecule was made via the optimization of PSCs with the functionalized MXene thin film employing three different organic molecules: Me‐4PACz, 2PACz, and H3pp, as shown in Figure S4a–c (Supporting Information). Our results indicate that the Me‐4PACz showed the best photovoltaic performance and device efficiency.

**Figure 2:**
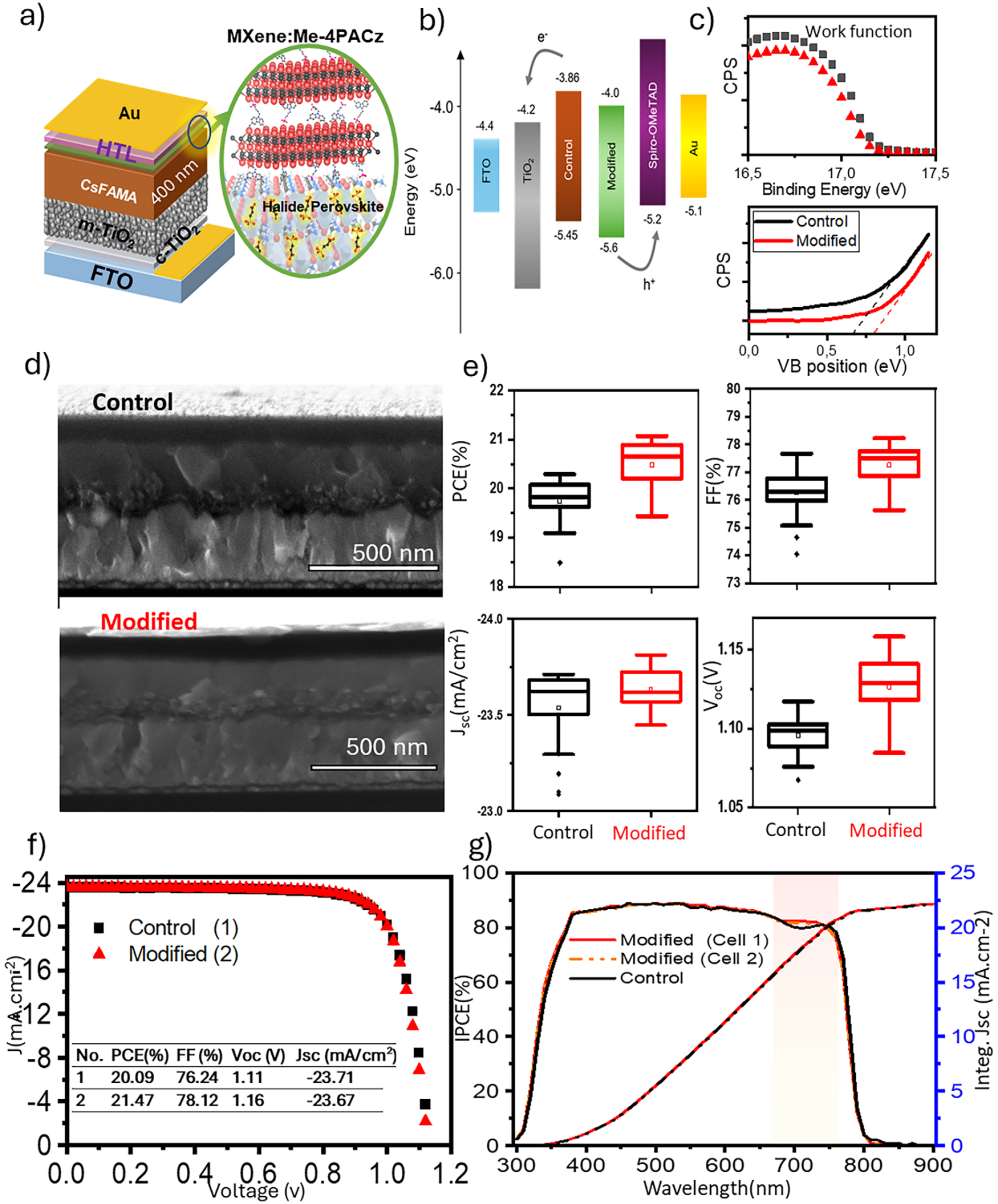
a) Schematic of device fabrication in normal configuration with CsFAMA perovskite film and Interface passivation using MXene:Me‐4PACz between perovskite/Spiro‐OMeTAD (HTL) layers, b) Energy band diagram calculated from UPS measurements and IPCE band gap estimation for devices with/without passivation (modified/Control) of perovskite film interface with MXene:Me‐4PACz, c) work function (WF) and Valence band (VB) position measurements of unmodified and modified perovskite films obtained from UPS analyses for the calculation of band diagram of the films, d) Cross‐section SEM images of the devices, e) the summary of the corresponding device parameters with/without passivation (modified/Control) of perovskite film interface with MXene:Me‐4PACz, f) J–V curve plots of the champion devices, g) IPCE spectra (left axis) and their corresponding integrated Jsc plots (right axis).

Thus, we selected this molecule to fabricate PSCs devices. Control perovskite solar cells were fabricated with the configuration FTO/c‐TiO_2_/m‐TiO_2_/CsFAMA/Spiro‐OMeTAD/Au (see details in Supporting Information). The modified PSCs were fabricated employing the MXene:Me‐4PACz as interface layer between the halide perovskite absorber and the HTL, having a final configuration FTO/c‐TiO_2_/m‐TiO_2_/CsFAMA/MXene:Me‐4PACz/Spiro‐OMeTAD/Au, were the perovskite absorber is passivated with the H3pp molecule as described in our previous work^[^ ^18^ ^]^ and in the Supporting Information. **Figure** **2**a shows a schematic representation of the PSC where the functionalized MXene:Me‐4PACz is located at the interface between the halide perovskite and the HTL, the Spiro‐OMeTAD.

Figure 2b shows the band diagram of the control and the modified PSCs with the Me‐4PACz molecule. The use of the functionalized MXene:Me‐4PACz thin film results in a lower valence band (VB), from −5.45 to −5.6 eV. Analogously, the conduction band (CB) is being reduced from −3.86 to −4 eV. The later values have been extracted from ultraviolet photoelectron spectroscopy (UPS) analysis of the samples, as shown in Figure 2c, where the work function and valence band positions are shifting to lower energies from the vacuum level and Fermi level, respectively, revealing of a shift downward in the band diagram. This band alignment indicates a more beneficial arrangement of the thin films, which allows for a more favorable charge injection from the CB of the perovskite into the CB of the TiO_2_. The presence of the MXene:Me‐4PACz at the perovskite/HTL interface reduces the VB position, preventing the hole recombination process occurring at the Spiro‐OMeTAD/perovskite interface. Thus, it can be inferred that MXene:Me‐4PACz passivation is creating a better stepwise energy diagram for hole transfer from the perovskite thin film into the HTL layer.

Figure 2d shows the corresponding cross‐section SEM images of the champion devices of the control and modified PSCs. As can be observed, the MXene:Me‐4PACz thin film forms a smooth and homogeneous layer between the halide perovskite and the Sprio‐OMeTAD thin film. Figure S5 (Supporting Information) shows the surface SEM images of perovskite film modified with MXene:Me‐4PACz, and Figure S6a,b (Supporting Information) show ED X‐ray spectrum and elemental mapping of a modified perovskite film with MXene:Me‐4PACz in analogy with the cross‐section SEM results.

The optimization of the photovoltaic parameters employing the MXene:Me‐4PACz (see Figure S7, Supporting Information) resulted in champion PSCs with superior properties to the control device, as shown in Figure 2e–g. The best photovoltaic parameters obtained were: 20.09% PCE, 1.11 Voc, 23.71 mA cm^−2,^ and 76.24 FF for the control devices, and 21.47% PCE, 1.16 V, 23.67 mA cm^−2^, and 78.12 FF for the MXene:Me‐4PACz device, as shown in Figure 2e,f.

Figure 2g shows the incident photon to current efficiency (IPCE) of the modified and control devices and the corresponding integrated Jsc values, which were 22.24 and 22.10 mA.cm^−2^ for the modified and the control PSCs, respectively. The latter is in good agreement with J–V measurements shown in Figure 2f. It is worth noticing that the modified device shows enhancement in IPCE spectra in the range of 682 to 740 nm from 79.7% to 82.5%.

In summary, the modified devices employing the MXene:Me‐4PACz thin film show enhanced photovoltaic performance demonstrated by electrical characterization (JV and IPCE plots) and by energy diagram and materials morphology.

**Figure 3:**
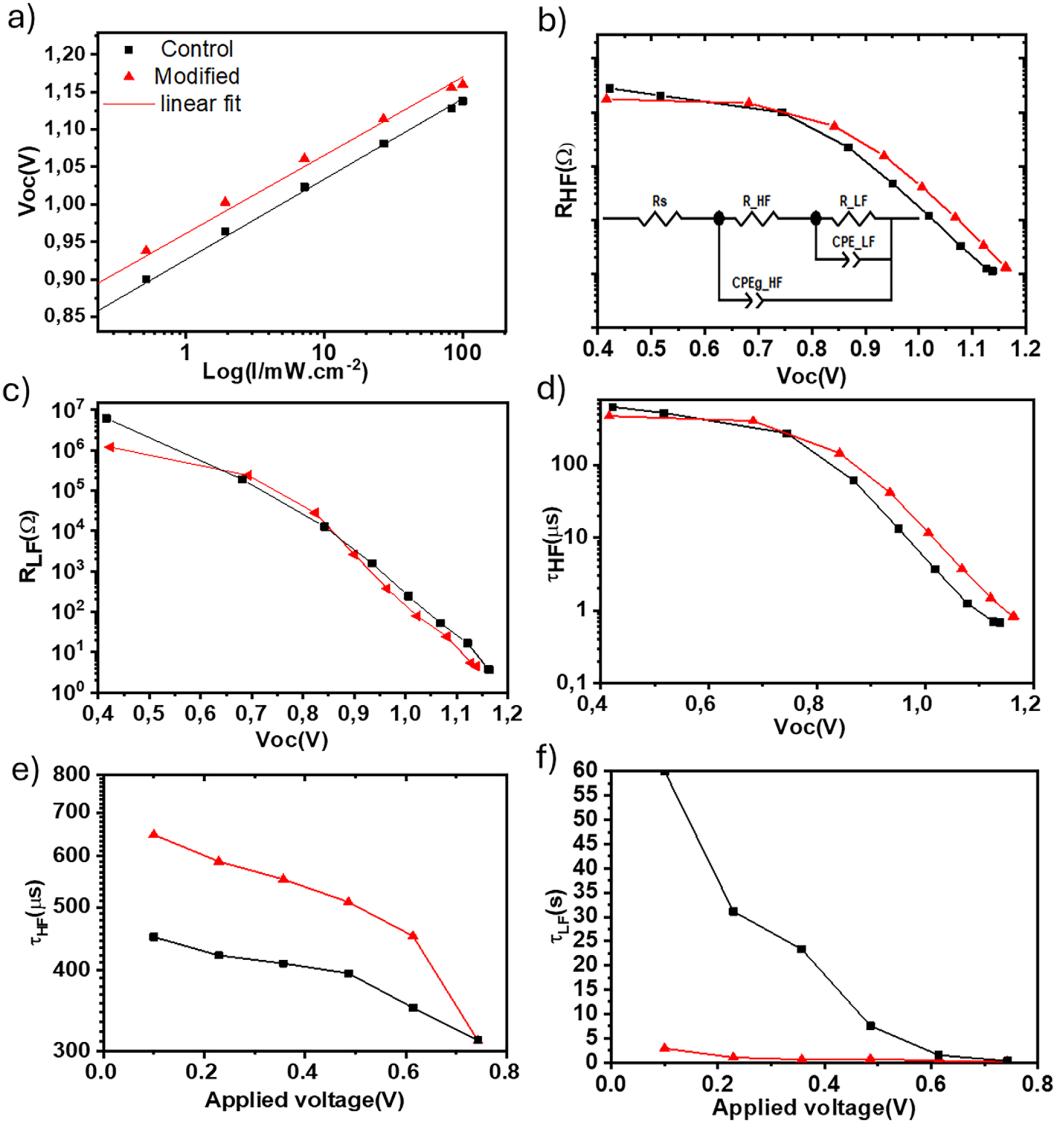
a) Voc‐Log Intensity profile of the devices for trap‐assisted recombination analysis, b,c) High frequency (HF) and low frequency (LF) resistance of the devices at different light intensities extracted from the simulation of Impedance spectroscopy (IS) with the equivalent circuit suggested in b. d) Carrier life time of the devices in HF region estimated from c. e,f) HF and LF carrier life time estimation extracted from simulated IS data under different applied voltages in dark conditions.

To understand the observed enhanced photovoltaic response for the as‐prepared PSCs with and without the MXene thin film, we carried out different electrical and optical characterizations. **Figure** **3**a shows the Voc‐Log intensity plot for the champion devices of each category. The obtained Ideality factor (n) from the linear fitting of the plots is 1.74, and 1.68 for the control, and the modified PSCs, respectively. Our results indicate that the modified device has a lower ideality factor and therefore shows less SRH recombination centers.^[^ ^40^ ^]^

Electrochemical impedance spectroscopy (EIS) analyses carried out for the devices at three different light intensities at Voc conditions, are shown in the Nyquist plots of Figure S8a–c (Supporting Information). The corresponding graphs obtained in dark conditions at different applied bias voltages to the devices are shown in Figure S8d–f (Supporting Information). Once collected, the EIS data were fitted by a standard model^[^ ^40^ ^]^ shown in the inset of Figure 3b. The electrical circuit is characterized by two parallel capacitor‐resistor branches connected in series. The first branch corresponds to high frequencies (HF), while the second is associated with low frequencies (LF). The former is mostly attributed to the response function of carriers (electrons and holes), while the latter one is attributed to the dynamic behavior of mobile ions in HP film.

Figure 3b,c shows the obtained resistance at HF and LF vs open circuit voltages at different light intensities. In general, we observed that all the devices reduce the total device resistance with the increase of light intensity, which is expected since more carriers are injected to the device with the increase of exposed light intensity.^[^ ^40^ ^]^ The reduction of semi‐circles in the Nyquist plots (Figure S8a–c, Supporting Information) are in an analogy to these results. It should be noted that generally these reductions in resistance do not imply better charge transfer properties of a specific device, but it can be inferred that all the devices show roughly the same R_HF_ values under very low light exposure (corresponding to low Voc). However, we also observed that for values above 0.75 V (corresponding to 10% 1‐Sun, see Figure 3b the results deviate from each other, with the modified PSC showing lower R_HF_. Therefore, the average lifetime at HF and LF regions was estimated using the following formula:

(1) τ=R.C

where R is the relevant real resistance and C is the capacitance obtained from the fitting.

Figure 3d shows the average lifetime in HF corresponding to the HF resistance plot in Figure 3c. It can be observed in both plots that the modified device behaves differently under low light and high light intensities if compared to the control PSC.^[^ ^18^ ^]^ At high Voc values, immobilizing the ions within their neighboring atoms can span the carriers’ lifetime, and their response under AC voltage is longer. Moreover, this immobilization can result in better charge transfer to the contacts and therefore increases the τ_HF_.^[^ ^41^ ^]^ Another approach in EIS is using only DC voltage while keeping the device in dark, so their AC response will be recorded at the presence of a constant electric field that could excite charge extraction and trap densities refilling. In impedance spectroscopy studies of perovskite solar cells under dark conditions with an applied DC voltage, at low applied voltages, the devices exhibit behavior dominated by shunt resistance. At higher applied voltages (close to the maximum power point (MPP) and beyond), the devices behave in a regime dominated by series resistance.^[^ ^40^ ^]^ Figure S8d–f (Supporting Information) shows examples of low voltage and high voltage Nyquist plots of the devices, and Figure 3e,f are the HF and LF lifetime of the devices resulted from the fitting of the Nyquist plots vs applied voltages in dark condition, respectively.

In summary, Figure 3e shows that in low applied voltages (similar to Shunt regime), the resistance and consequently the τ_HF_ for Modified devices are higher, as inferred also by Figure S8d (Supporting Information).^[^ ^40^ ^]^ However, when the applied voltage is increased (Figure S8e,f, Supporting Information), both modified device's resistance and τ_HF_ decreased rapidly and became comparative with the control device. More interestingly, the evolution of τ_LF_ in Figure 3f is very interesting. While the τ_LF_ for the control PSC shows high values at low voltages and decline with the increase of applied voltage, the modified PSC shows much lower values across the same voltage range. This response could be attributed to the efficient immobilization of ions inside the modified PSC. Therefore, in the shunt regime for τ_HF_, the carrier lifetime can be interpreted as the recombination resistance enhancement and in higher applied voltages, the rapid reduction of τ_HF_ could be assigned to better charge transfer at the contacts due to passivation of perovskite film.

**Figure 4:**
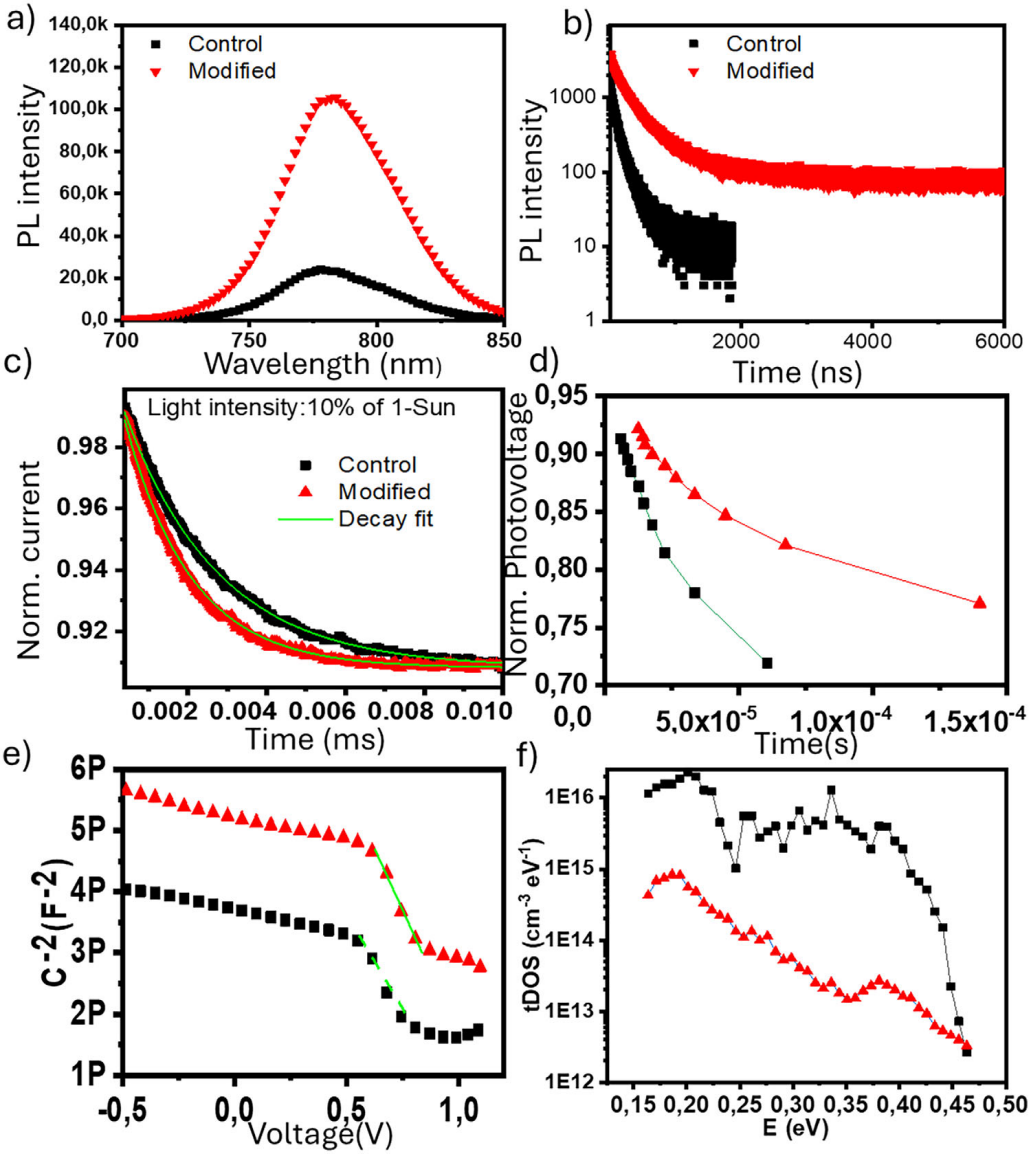
a) Photoluminescence (PL) b) Time‐resolved Photoluminescence (TRPL) spectra of the perovskite films with/without passivation on Glass substrates, c) Normalized Transient photocurrent decay spectra of the devices under 10% of 1‐sun irradiation, d) Normalized photovoltage decay measurements, e) Mott–Schottky plots, f) Trap density vs energy profile from the conduction band of the devices.

To further investigate the comparative passivation mechanisms between the devices, versatile experiments were conducted to reveal more facts about the correlation of the stability of the devices with the passivation of trap states in the bulk and at the interface of HP film. **Figure** **4**a,b shows the Photoluminescence (PL) and time‐resolved Photoluminescence (TRPL) spectra of the films deposited on glass with and without passivation, respectively. PL intensity increases as passivation proceeds for the Modified film, respectively. Such order of enhancement is also observed exactly in TRPL results as it indicates the passivation of non‐radiative centers.^[^ ^16^ ^]^ But solely this proof of passivation cannot be interpreted as it can lead to a higher charge extraction rate in passivated films, where there are some types of trap states that are radiative active in PL spectroscopy.

Hence, Transient photocurrent (TPC) spectroscopy was performed for the devices where a relative intensity pulse of light illuminates the device and excites the carriers, and the current decay signal after the light‐off is recorded to elucidate how fast charges are extracted at the contacts. Figure 4c shows the TPC spectra for 10% of simulated 1‐Sun intensity (LED source), respectively. Interestingly, the order of short decay time (τ_TPC_) for the fitted curves in Figure 4c are 16, and 24 µs for the Modified, and Control devices, respectively. It clearly indicates a faster charge extraction for the passivated device in well agreement with the PL and TRPL analyses.

Another proof for extending carrier lifetime and better charge extraction is transient photovoltage decay (TPV) when a pulse of light is imposed on the devices and the capacitive charge decay behavior in open circuit conditions is studied.^[^ ^15^ ^]^ A device with less trap states will hold the voltage across the cell for longer periods of time. Thus, a PSC with more passivated defects will show a longer the decay.^[^ ^15^, ^40^ ^]^ In view of this, from Figure 4d the order of better performance devices is in accordance with TPC and TRPL analyses.

To quantitatively calculate the number of trap densities for the devices (please see Supporting Information file for the calculations), Mott–Schottky analysis was performed at 10 kHz, with different off‐set potentials, and the results are shown in Figure 4e. The calculated trap density (N_D_) is 1.62 × 10^16^ and 2.2 × 10^16^ cm^−3^ for the modified and control devices, respectively, an indication that ≈30% of the bulk defects have been passivated. To delve on the energy dependent of the trap state passivation, we performed a series of Thermal Admittance Spectroscopy (TAS) and the results of trap density of states of selected devices are shown in Figure 4f. We can observe a significant reduction of both shallow and deep trap states across all the range of energies up to 0.45 eV below conduction band (CB) of HP for the Modified device compared to the Control. The modified device shows a significant decrease in the trap densities by a factor of more than 10–1000 times across the energy profile scan.

In summary, the in‐depth analyses show that the application of the functionalized MXene can enhance charge extraction in the PSC through improved band alignment and passivated deep and shallow traps, if compared to the control (non‐modified) device. The later induces significant hydrophobic properties of perovskite film that can incorporate constructive in its enhanced outdoor operational stability.^[^ ^16^ ^]^

**Figure 5:**
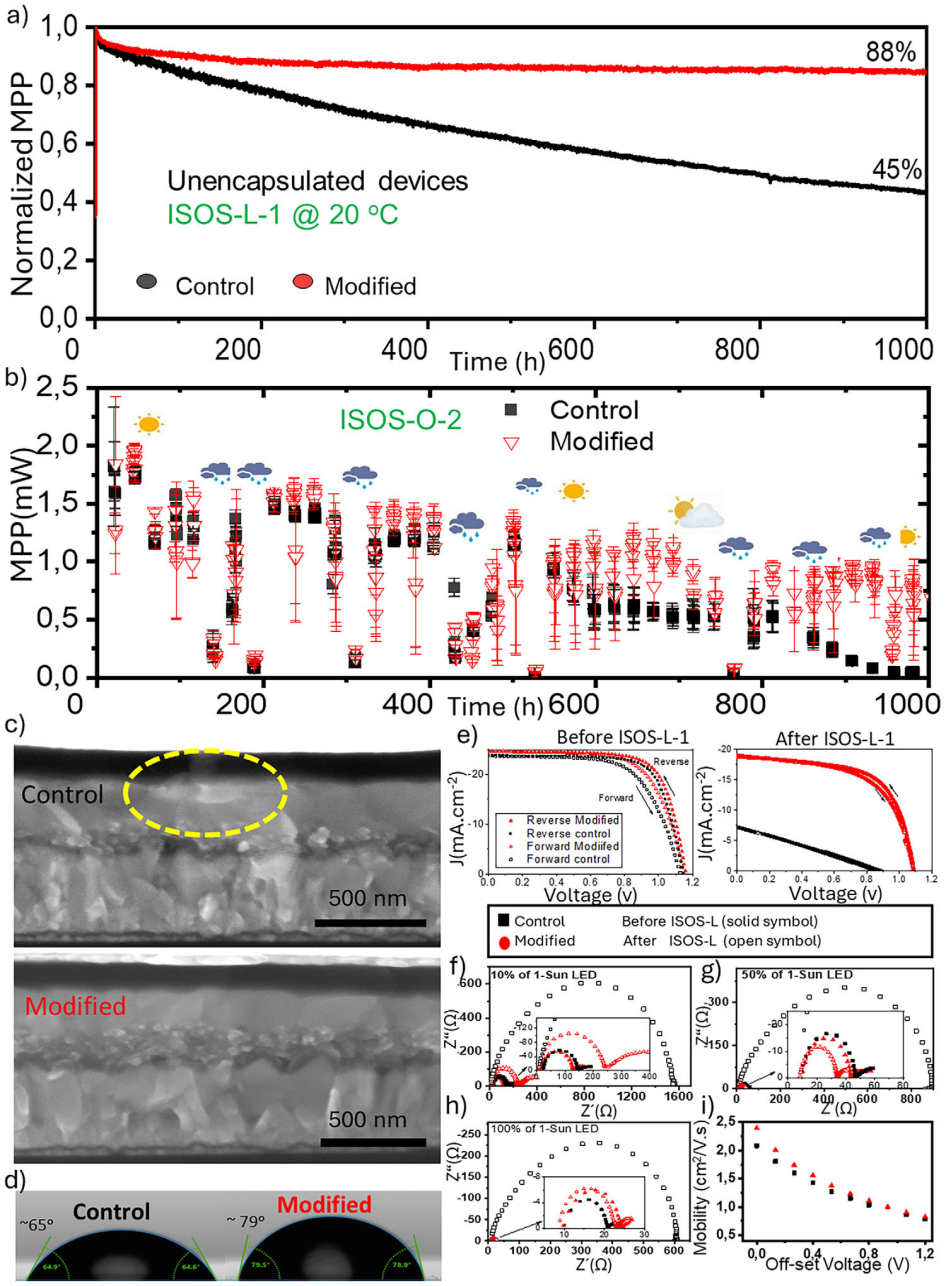
a) Normalized averaged indoor stability assessment of devices (ISOS‐L‐1) by tracking their MPP under continuous 1‐sun irradiation @ 20 ^o^C, and under N_2_ flow for 1000 h. b) Averaged Maximum MPP evolution of the devices in outdoor stability assessment by holding encapsulated devices at their open circuit voltage and recording their I–V curves with 10 min time intervals for 1000 h. (University of Autonoma of Barcelona, 41.5021° N, 2.1039° E, Spain). c) Cross‐section SEM of the devices after ISOS‐L‐1 assessment, d) Contact angle measurements of the films without passivation (Control) and with passivation of perovskite film (Modified). e) J–V curves recorded Before and After ISOS‐L‐1 assessment for the same champion devices. f–h) Nyquist plots of the champion devices before (solid symbols) and after (open symbols) ISOS‐L‐1 protocol at 10%, 50%, and 100% of simulated 1‐sun, respectively. i) Carrier mobility vs applied voltage extracted from CELIV measurements for champion devices after ISOS‐L‐1 protocol.

To demonstrate the beneficial effect of the interface modification employing the Me‐4PACz functionalized MXene, we carried out stability analyses of the modified PSCs and compared them with the control devices without modification. **Figure** **5**a,b shows the indoor and outdoor stability analyses, respectively, carried out to a selected set of devices (between 4 and 6).

Figure 5a shows the indoor stability assessment under continuous light soaking (ISOS‐L‐1) carried out under N_2_ atmosphere and at room temperature. Our results indicate enhanced stability for the modified PSCs in comparison with the control devices.

After 1000 h the modified devices show a retention of 88% of their initial MPP value (T_88_ = 1000 h), while the control device showed only a 45% (T_45_ = 1000 h), which means that our passivation strategy has prolonged almost twice the stability of the 3‐cation perovskite solar cells.

We also carried out outdoor stability analysis following the ISOS‐O‐2 protocol in Figure 5b. For this, the devices were encapsulated using a glass‐to‐glass encapsulation employing epoxies as described in detail in our recent report.^[^ ^42^ ^]^ The outdoor analysis was carried out holding devices at their open circuit voltage (Voc) and recording their daily IV curves, which is a more aggressive stability assessment methodology than the classical MPP tracking applied for PSCs.^[^ ^43^ ^]^ Figure 5b illustrates the overall outdoor stability of the devices (3 and 4 devices per category) by indicating the extracted average values of maximum power point evolution during a period of 1000 h. We can observe that the control devices degraded completely after 1000 h (a T_50_ = 550 h) while the modified PSCs show more than 50% of their initial output power after the same period under analysis (T_50_ = 1000 h). The latter demonstrates the beneficial effect of the functionalized MXene as part of the HTL in our PSCs.

Although the comparison between the stability under both conditions (indoor and outdoor) is not straightforward, we can observe that for the same type of PSCs, a better stability is observed under indoor conditions. The outdoor analysis can be considered more aggressive than the indoor, as reflected by the humidity, and input power variations observed under outdoor tests as shown in Figure S9a (Supporting Information). Under ISOS‐O‐2 the PSCs undergone harsh environmental conditions, with fluctuations in many environmental parameters such as temperature, humidity, light irradiation, among others. The latter in addition to aggressive intrinsic stressor (applied bias at Voc at all times).

Interestingly, the PSCs analyzed outdoors demonstrated a nice PCE recovery after being subjected to intense rainy days and/or low light irradiation conditions (for example as observed at ≈400 and 550 h), after which both PSCs recovered their PCE. After these recoveries, we observed that the control PSCs degrade faster than the modified PSC. Thus, the passivation with functionalized R‐MXene results in spanning the outdoor lifetime of the devices roughly twice as long. Figure S9b–g (Supporting Information) shows the daily evolution of the MPP of the devices for selected days to inspect the daily performance of the devices especially in full sunny days (Figure S9a (Supporting Information); reaching ≥ 1000 W m^−2^), and rainy days (solar irradiance ≈< 150 W m^−2^).

As it is indicated at the beginning of experiment up to 500 h, all the categories are functioning and approaching almost the same MPP (Figure S9b,c, Supporting Information). But in a cloudy day after 520 h, Figure S9d (Supporting Information), the Control devices show lower MPP while Modified one reached roughly the 4 times MPP values. Figure S9d–g (Supporting Information) indicate the gradual degradation of the control devices, while the performance of the modified PSCs is much better, retaining the output power almost completely. It should be noted that in Figure S9f (Supporting Information), in low light irradiance (maximum input power of the day was 10% of 1‐Sun, 100 W m^−2^), the modified has a maximum MPP value of 0.84 mW which is roughly 4 times of the control devices. In Figure S9g (Supporting Information), the control devices have degraded almost completely, while the modified PSCs retain 50% after 1000 h.

To further understand the reasons behind the stability enhancement observed for the PSC employing the MXene layer, we carried out cross‐section SEM, and contact angle (CA) measurements to the samples. Figure 5c shows the cross‐section SEM analysis of the control and modified PSCs after 1000 h under the light soaking experiment (ISOS‐L‐1). The images show a clear difference on the materials after testing. Materials segregation is observed at the interface between the perovskite and the Spiro‐OMeTAD, specially there is clear evidence of deterioration of the Spiro‐OMeTAD layer in the control PSC which is not observed for the modified device, as indicated by the dashed yellow circle of Figure 5c, see also Figure S10 (Supporting Information). The latter corroborate that the halide perovskite degradation is one of the causes of the deterioration of the PSC performance for the control PSC.^[^ ^40^ ^]^

To investigate also the effect of the MXene thin film on the crystal structure and texturing of halide perovskite thin film, XRD data for the films were collected as shown in Figure S10b,c (Supporting Information). Our results indicate that the MXene‐modified thin film shows a decrease in the PbI_2_ peaks and an improved crystallinity of the perovskite cubic phase, as shown in Figure S10c (Supporting Information). The latter demonstrates that the presence of the MXene benefits the halide perovskite crystallinity and prevents or delays the formation of PbI_2_.

CA measurements are shown in Figure 5d. Our results show an enhancement of the hydrophobic properties of the perovskite thin film when the MXene:Me‐4PACz is applied, observing a 64–65° angle for the control PSC and an increase to 79–80° for the modified PSC. Thus, it can be inferred that the presence of the MXene: Me‐4PACz thin film enhances the hydrophobicity of HP film.

To further understand the charge transport evolution of the PSCs, we carried out a series of impedance spectroscopy analyses and charge extraction measurements under light induced voltage (CELIV) for both control and modified PSCs before and after the 1000 h of ISOS‐L‐1 assessment. Figure 5f,h show the comparison of the Nyquist plots obtained from the IS measurements for the same devices at open circuit voltage under 10%, 50% and 100% of 1‐Sun illumination, respectively, before (solid symbols) and after (open symbols) the ISOS‐L‐1 stability assessment. In general, we observed that the control PSC shows a significant increase of impedance after the light‐soaking stability (ISOS‐L‐1) experiment, which clearly is related to the degradation of the perovskite film. These results are in good agreement with the cross‐section SEM images shown in Figure 5c and in agreement with the JV curves before and after ISOS‐L‐1 stability shown in Figure 5e. The J–V curves observed after stability assessment for the control (black curve) device shows a significant deterioration of device parameters in comparison with the J–V curves of the modified PSC (red curve).

In Figure 5f, We also observed that at low light exposure (10%), the modified PSC shows roughly 2 folds increase of their resistance, while it shows also a clear deformed semicircle at low frequencies with low imaginary admittance values and large real impedance values. Figure 5f ‐inset, the Modified device shows a decrease in the charge transfer resistance even after ISOS‐L assessment in both 50%, and 100 % light exposures, which could be due to slight perovskite degradation and the increase of carrier recombination at the interface. These results demonstrate the enhancement on the charge transfer processes observed in the modified PSC after 1000 h of continuous light‐soaking analysis. Finally, to reveal this enhancement, the mobility of the devices was obtained using CELIV measurements for different off‐set voltages, and the results are plotted in Figure 5i. It clearly indicates the enhanced mobility of the Modified device compared to the Control device across all the applied voltages.

In summary, the application of functionalized MXene:Me‐4PACz thin film employed as part of the HTL in PSCs as an interface between the HTL and the halide perovskite resulted in higher operational stability of the devices, both from indoor and outdoor stability assessments. These results demonstrate that the Me‐4PACz molecule permits the enhancement of the PSCs at low‐light conditions, observing better charge extraction abilities.

**Table** **1** summarizes the comparisons of different studies using MXenes nanomaterials application/processing in Lead‐based Perovskite device fabrications, with specific indication of their exact role in the device configuration to promote their performance as well as their operational stability. From the configurational point of view and type of passivation, the most significant difference between the current study and other reports is that our study is employing the MXenes at the interface of Hole transport layer (HTL)/Halide perovskite (HP) while other reports are emphasizing either employing it at perovskite/Electron transfer layer (ETL) interface or in mixed with HP film to control the grain growth and boundary defects. In addition, there are seldom reports that employing functionalized MXenes in the device fabrication using organic ligands to modify interlayer spacing of the MXenes,^[^ ^16^, ^44^ ^]^ while in this report we have shown direct observation of interlayer functionalization of MXenes with a hole extracting molecule using STEM‐EELS characterization. Moreover, comparing different MXenes processing revealed that we have employed a novel delamination process for obtaining the MXene nanomaterials for the current study. In addition to that, in Table 1, our report is the only study indicate of both indoor and real outdoor stability study of MXene based perovskite solar cells that indicated enhanced outdoor stability up to 1000 h, which is superior also to our previous report of 600 h.^[^ ^16^ ^]^ Comparison of indoor stability studies in Table 1 shows our study is the topmost enhanced indoor stability using continuous light soaking conditions and MPP tracking (ISOS‐L‐1) with T_88_@ 1000 h among all triple‐cation CsFAMA based perovskite solar cells such as the report of Zhao et al.^[^ ^32^ ^]^ with T_70_ and T_81_ @ 1000 h.

**Table 1:** Comparison of PSCs’ performance and stability fabricated using MXene nanostructures.

MXene	HP	Conditions	MXene Application in Device	Device Parameters [%]	Atmosphere	Stability Protocol^*^	Stability Results	Refs.
Ti_3_C_2_T_X_	CsPbI_3_	LiF/HCl mixture	Oxidized Ti_3_C_2_T_X_ as additives for PVK	V_OC_: 1.18–1.21, J_SC_: 19.05–19.86, FF%: 80.33–81.96, PCE%: 18.06–19.69	N_2_	ISOS‐L‐2 @85 ^o^C	T80 @ 1000 h	[45]
Ti_3_C_2_T_X_	CsFAMA	LiF/HCl mixture	SnO_2_‐MXene for ETL	V_OC_: 1.09–1.13, J_SC_: 24.16–25.07, FF%: 75.8–81.1, PCE%: 20.03–23.07	N_2_	ISOS‐D‐1	T90@500 h	[46]
Ti_3_C_2_T_X_	FA_0.9_MA_0.05_Cs_0.05_PbI_0.98_Br_0.02_	Purchased	MXene quantum dot‐ SnO_2_ of ETL	V_OC_: 1.140–1.172, J_SC_: 24.26–24.96, FF%: 75.8–79.8, PCE%: 20.96–23.34	N_2_	ISOS‐L‐1	T90@500 h	[47]
Ti_3_C_2_Cl_X_	FA_0.85_MA_0.15_Pb(I_0.95_Br_0.05_)_3_	Purchased	Ti_3_C_2_Cl_X_ for SnO_2_ ETL	V_OC_: 1.173–1.203, J_SC_: 24.81–25.16, FF%: 78.05% to 82.14%, PCE%: 22.72–24.86	Air	ISOS‐D‐3 @85 ^[o]^C	T80 @ 1002 h	[31]
Ti_3_C_2_T_2_ and Nano‐Ti_3_C_2_T_X_	FAPbI_3_	HF method and Pulsed Laser irradiation	Adding into antisolvent for PSK interfacial embedding	V_OC_: 1.102–1.132, J_SC_: 25.30–25.84, FF%: 79.81–82.63, PCE%: 22.25–24.17	N_2_	ISOS‐L‐1	T90@1000 h	[30]
Nb_2_C	Cs_0.05_FA_0.88_MA_0.07_PbI_3_	Solvothermal method	SnO_2_‐Nb_2_C ETL	V_OC_: 1.111–1.138, J_SC_: 24.71–25.29, FF%: 69.1–79.5, PCE%: 18.96–22.86	N_2_	ISOS‐D‐1	T98@ 40 days	[48]
Ti3C2Tx QDs	CsFAMA	LiF/HCl mixture	mTiO2‐TQD/TQD‐perovskite	V_OC_: 1.10–1.13, J_SC_: 23.11–23.64, FF%:71.83–77.56, PCE%: 18.26–20.72	N_2_	ISOS‐L‐1 under UV 365 nm light source	T90 @200 h	[49]
Ti_3_C_2_T_X_	(FAPbI_3_)_0.97_(MAPbBr_3_)_0.03_	LiF/HCl mixture	SnO_2_‑MXene ETL	V_OC_: 1.07–1.10, J_SC_: 21.88–22.03, FF%: 71.89‐77.78, PCE%: 16.83–18.90	N_2_/air	ISOS‐D‐1	T85@45 days	[50]
Ti_3_C_2_T_X_	(FAPbI_3_)_X_(MAPbBr_3_)_1−X_	LiF/HCl mixture	Interlayer (MXene‐CNTs) between the ETL and PSK	V_OC_: 1.043–1.073, J_SC_: 24.77–25.09, FF%: 0.73–0.80, PCE%: 18.84–21.42	N_2_	ISOS‐L‐1	T55@300 min	[51]
Ti_3_C_2_T_X_	CsFAPbI_3_	LiF/HCl mixture	MXene composites to BCP for ETL	V_OC_: 1.00–1.02, J_SC_: 21.75–22.31, FF%: 76–76, PCE%: 16.45–17.46	N_2_	ISOS‐L‐2 @85 ^[o]^C	T96@2300 h	[52]
Ti_3_C_2_T_X_	(FAPbI_3_)_0.95_(MAPbBr_3_)_0.05_	Purchased	SnO_2_‐MXene ETL	V_OC_: 1.119–1.138, J_SC_: 24.49–24.58, FF%: 77.18–79.99, PCE%: 21.17‐22.38	N_2_	ISOS‐D‐3 @85 ^[o]^C	T80@300 h	[53]
Ti_3_C_2_Cl_X_	Rb_0.05_Cs_0.05_(FA_0.83_‐MA_0.17_)_0.90_Pb(I_0.83_Br_0.17_)_3_	LiF/HCl mixture or HF method	As additives for PSK	V_OC_: 1.14–1.19, J_SC_: 21.16–22.27, FF%: 77.85‐80.4, PCE%: 18.78–21.31	N_2_	ISOS‐D‐1	T84@1000 h	[54]
Ti_3_C_2_T_X_	Cs_0.05_(FA_0.85_MA_0.15_)_0.95_Pb(I_0.85_Br_0.15_)_3_	HF/HCl mixture	Ti_3_C_2_T_X_ as additive of PSK	V_OC_: 1.157–1.181, J_SC_: 23.25–23.50, FF%: 74.83–77.59, PCE%: 20.14–21.52	N_2_	ISOS‐L‐1	T81@1000 h	[32]
Ti_3_C_2_T_X_	(FAPbI_3_)_0.85_ (MAPbBr_3_)_0.15_	HF method	TiO_2_:MXene ETL	V_OC_: 1.178–1.091, J_SC_: 25.58–25.41, FF%: 81.71–77.15, PCE%: 24.63–21.38	Ambient	ISOS‐D‐1	T100@ 7 weeks (49 days)	[55]
Ti_3_C_2_T_X_	FAPbI_3_	LiF/HCl mixture	Functionalized MXene modify SnO_2_ for ETL	V_OC_: 1.062–1.118, J_SC_: 25.01–25.44, FF%: 79.03–83.18, PCE%: 20.98–23.66	N_2_	ISOS‐L‐1	T60/T80@1000 h	[44]
Mo_2_CTx	HP	HF/TBAOH	Perovskite/MXene mixture	PCE 24.05%	N_2_	ISOS‐D‐1	T89@3000 h	[56]
Ti_3_C_2_T_X_	CsFAMA	New delamination	HTL/HP interface	V_OC_: 1.19 J_SC_: 24.86, FF%: 81.29, PCE: 21.5%	N_2_	ISOS‐L‐1	T88@1000 h	This work
					Encapsulated	ISOS‐O‐2	T50@1000 h	

*Please refer to the Ref.^[^ ^43^ ^]^ for the details of stability protocols.

## 3 Conclusion

In this work, we successfully delaminated and functionalized the Ti_3_C_2_ MXene with the Me‐4PACz molecule. The intercalation of the organic molecule within the MXene labs was confirmed by HRTEM‐EELS analyses.

The MXene:Me‐4PACz was applied as interfacial passivation layer between the HP and the Spiro‐OMeTAD (HTL) thin film, resulting in a homogeneous thin film with nanoneedle‐like morphology at the surface. Complete PSC devices were fabricated and characterized, as well as its indoor and outdoor operational stability. The result shows that the application of the MXene:Me‐4PACz in PSCs enhances the indoor and outdoor operational stability. We observed that this resultant passivation improved the operational indoor stability (ISOS‐L‐1) by retaining 88% of initial MPP values after 1000 h of continuous light irradiation, while the control device lost more than 40–50% of their initial MPPs. The outdoor stability assessment results indicate a significant improvement of the stability retaining 50% of the initial efficiency after 1000 h for the Modified device, while the control devices degraded completely after 1000 h. Different characterizations revealed the reason for the operational stability enhancement is improved band energy alignment, simultaneous shallow and deep trap states passivation and charge extraction enhancement. Cross‐SEM, J–V measurements and Impedance spectroscopy studies before and after indoor stability assessment revealed that the degradation suppression is mainly due to ion migration suppression toward the interface that leads to retaining device parameters and operational performance of the Modified device using MXene:Me‐4PACz.

## Conflict of Interest

The authors declare no conflict of interest.

## Supporting information



Supporting Information

## Data Availability

The data that support the findings of this study are available from the corresponding author upon reasonable request.
