# Supplementary material for: Me‐4PACz Functionalized MXene for Halide Perovskite Solar Cells
Source: Adv Sci (Weinh). 2025 Aug 14;12(39):e09898. doi: 10.1002/advs.202509898 (PMC12533403; doi:10.1002/advs.202509898)
Supplement: Supplementary file 1 — Supporting Information [file ADVS-12-e09898-s001.docx]

Supporting Information

Me-4PACz-functionalized MXene Nanoneedles for Halide Perovskite Solar Cells

*Masoud Karimipour,^*a^ Nil Monrós Oliveras,^a^ Zhenchuan Tian,^a^ Francesco Salutari,^a^ Maria Chiara Spadaro,^a,b^ Tiankai Zhang,^c^ Naji Vahedigharehchopogh,^a^ Jordi Arbiol,^a,d^ Feng Gao^c^ and Monica Lira-Cantu^*a^*

^a^ Catalan Institute of Nanoscience and Nanotechnology (ICN2), CSIC and BIST, Campus UAB, Bellaterra, 08193 Barcelona, Catalonia, Spain

^b^ Department of Physics and Astronomy “Ettore Majorana”, University of Catania and CNR-IMM, 95123 Catania, Italy

^c^ Department of Physics, Chemistry and Biology. (IFM), Linköping University, Linköping, 58183, Sweden.

^d^ ICREA, Pg. Lluís Companys 23, 08010 Barcelona, Catalonia, Spain

E-mail: masoud.karimipour@icn2.cat, monica.lira@icn2.cat

Table of contents

[1. Materials and methods 1](#_Toc203312123)

[2. Device fabrication 2](#_Toc203312124)

[3. Device encapsulation for outdoor stability tracking tests 3](#_Toc203312125)

[4. Characterization tools 3](#_Toc203312126)

[5. Initial assessment of device fabrication using MXene: Me-4PACz as interfacial passivating layer 6](#_Toc203312127)

[6. Supplementary Figures and Tables 7](#_Toc203312128)

# Materials and methods

- 1. **Materials for perovskite layer, interface modification layer, and hole transport layer:** Lead(Ⅱ) bromide (PbBr_2_, 99.99%), lead(Ⅱ) iodide (PbI_2_, 99.99%), cesium iodide (CsI_2_, 99.99%), rubidium iodide (RbI_2_, 99.99%), formamidinium iodide (FAI), methylammonium iodide (MAI), and [4-(3,6-Dimethyl-9H-carbazol-9-yl) butyl] phosphonic acid (Me-4PACz) were acquired from Tokyo Chemical Industry (TCI) Co. Ltd. The hole transport layer material, Spiro-OMeTAD, was purchased from Xi’an Polymer Light Technology Co. 4-tert-Butylpyridine (t-BP), tris(2-(1Hpyrazol-1-yl)-4-tert-butylpyridine) cobalt (III) tri[bis-(trifluoromethane) sulfonimide] (FK209), Lithium bis(trifluoromethanesulfonyl)imide (Li-TFSI) were obtained from Aldrich. Dimethyl sulfoxide (DMSO), N, N-dimethylformamide (DMF), chlorobenzene (CB), acetonitrile (CAN), isopropanol (IPA), and 4-tert-butylpyridine (TBP) were provided by from Merck Chemicals Co. Ltd. Tetrapropylammonium hydroxide (TPAOH), tetrabutylammonium hydroxide (TBAOH), tetramethylammonium hydroxide (TMAOH) and 3-phosphonopropionic acid (H3pp) were obtained from Sigma-Aldrich. Ti_3_C_2_T_X_ nanosheet powder was supplied by Nanochemazone Inc. All materials were used as received unless specified otherwise.
  2. **Delamination process of Ti_3_C_2_T_X_ nanosheets and their functionalization with Me-4PACz (MXene:Me-4PACz nano flakes):** Blending 10 mg Ti_3_C_2_T_X_ powder with 3 mL of TPAOH and 1mL of Hydrazine hydrate and stirring at ambient temperature for 24 hours, following that, TBAOH and TMAOH were used to treat Ti_3_C_2_T_X_ powder subsequently and stir it overnight each separately. The final powder was firstly centrifuged 1000 rpm for 30 sec and then the green supernatant was collected and centrifuged with 12000 rpm for 30 min, then it was washed with IPA and centrifuging three times with the same speed, re-dispersed in 3 mL of IPA to acquire the dark green color solution and stored in the refrigerator for future use. A specific quantity (1 mg and 2 mg) of [4-(3,6-Dimethyl-9H-carbazol-9-yl) butyl] phosphonic acid (Me-4PACz) were added to 2 mL of previous delaminated Ti_3_C_2_T_X_ solution and treated by bath ultrasonication for 20 min.
  3. **Preparation of perovskite precursor solution and the Modified perovskite:** Cs_0.05_(FA_0.85_MA_0.15_)_0.95_Pb(I_0.9_Br_0.1_)_3_ precursor solution was prepared by firstly, PbI_2_/PbBr_2_ precursor solution was prepared by dissolving 548.6 mg/ 57.06 mg of PbI_2_/PbBr_2_ in 1mL of DMF:DMSO (4:1 volume ratio) at 150ºC for 5-6 min, then allowed to cool down. Thereafter, it was injected to a vial containing 27.02 mg of CsI, 178.94 mg of FAI, and 17.41 mg of MABr and was shaken for 20 sec. and dissolved completely at 75ºC for 15 minutes to form the tripl/cation perovskite Cs_0.08_MA_0.12_FA_0.8_Pb(I_0.88_Br_0.12_)_3_. For H3pp bulk passivation (H3pp:HP), 4.5 µl of H3pp stock solution (20 mg/ 0.5mL DMF) was injected into 0.5 mL of triple-cation perovskite solution and was shaken for 20 sec.
  4. **Preparation of hole transport layer solution, Spiro-OMeTAD:** 60 mg of Spiro-OMeTAD was dissolved in 0.59 mL chlorobenzene. Then, blending 23 µL of t-BP, 4.5 µL of FK209 (375 mg in 1 mL acetonitrile), and 13.5 µL Li-TFSI precursor solution (100 mg in 378 mL acetonitrile) with previous solution before deposition.

# Device fabrication

Patterned FTO (16 Ω/sq, 2.5 cm*1.5 cm) coated onto glass substrates were washed with 10% Hellmanex solution firstly, then cleaned by bath ultrasonication with 2% Hellmanex solution, acetone, and isopropanol for 30 min, 10 min, 15 min subsequently. Afterwards, the substrates were treated with UV/Ozone for 20 min and placed on hotplate at 450 ℃ for following TiO_2_ blocking layer deposition. Then, the dense TiO_2_ precursor solution, comprising of 0.4 mL of acetyl acetone, 0.6 mL of Titanium diisopropoxide bis-acetyl acetonate, and 9 mL of ethanol, was sprayed onto the preheated substrates for 6-8 min, followed by maintaining them at 450 °C for 30 min. After cooling down to ambient temperature, the mesoporous TiO_2_ solution, made by mixing 1 mg of TiO_2_ nanoparticles (30 nm) and 6 g of ethanol thoroughly, was spin coated on the substrates at 5000 rpm for 20 s, then dried and annealed at 80 °C, 450 °C for 30 min, respectively, to form the electron transport layer (ETL). Before perovskite layer, interface modification and hole transport layer deposition, the substrates were exposure to UV/Ozone for 25 min and moved directly to the nitrogen-filled glove box (Temperature: 18-20 ℃, O2: 3-6 ppm, H_2_O: 1-1.5 ppm).

The perovskite films were fabricated by one-step deposition. Firstly, 50 μL perovskite precursor solution (or **bulk modified perovskite with H3pp**) was spin coated onto the substrates at 2000 rpm for 10 s and 5000 rpm for 30 s, chlorobenzene (CB) was used as anti-solvent in the last 15 s, then the film was annealed at 110 °C for 45 min. The **MXene:Me-4PACz nano needles** surface modification of Modified HP thin films were performed using drop casting of 55 μL of the ink solution (3mg/mL concentration) on the top of perovskite film at 3000 rpm, 20 sec. and annealed at 85 °C for 3-5 min. The resultant device is recalled as **Modified** in the entire manuscript**.** Thereafter, the hole transport layer was deposited onto the above passivation layer by spin coating 45 μL Spiro-OMeTAD solution at 3800 rpm for 20 s. Finally, 80 nm Au electrodes were evaporated thermally on the top of HTL by high vacuum PVD system with pressure of 8*10^-7^ atm for 1 h.

# Device encapsulation for outdoor stability tracking tests

Solar cells were encapsulated using a pristine glass stick to the cells with a UV curing epoxy. After 2 hours of UV curing, the edges of the cells were sealed using a bi-component epoxy to completely isolate the cell from the O_2_ and humidity from the atmosphere. A tape with a rounded area of 0.160 cm^2^ was attached on the sun-exposed side of the cell to mark out the irradiated area for the Jsc values. Once PSC were encapsulated, they were brought to the roof of ICN2 (Bellaterra, Barcelona) where there is a well-equipped outdoor tracking system. Reverse scans from 1.2 to -0.1 V were performed every 10 minutes.

# Characterization tools

Current Density-Voltage (J–V) measurements were performed using a HelioSim-CL60 solar simulator from Voss electronic GmbH with a source meter (Keithley 2400) in the ambient atmosphere under 1 sun illumination (AM 1.5 G) at room temperature. 0.160 cm^2^ aperture area metal mask was used to define the active area. Reverse scans from 1.2 V to 0 V in all devices were set to a scan speed of 100 mV/s. Incident photon to current conversion efficiency (IPCE) were measured by a power source (300 W xenon lamp) with a commercial apparatus (Arkeo-Ariadne from Cicci Research) from 300 to 900 nm wavelength. Photoluminescence (PL) spectra of the samples have been obtained by a PicoQuant system that offers both excitation and emission wavelength scans. The sample can be excited by a pulsed laser, LED, or Xe-flash lamp. The morphology of the products has been examined using a field emission scanning electron microscope (FEI Quanta 650 FEG ESEM), which is suitable for all types of samples (conductive, insulating, and wet materials). The applied acceleration voltage was 10 kV, and the base pressure was 1.5×10^-5^ Torr. The instrument has three detection systems: for secondary electrons, for back-scattered electrons, and for transmitted electrons. In addition, Energy-Dispersive X-Ray Spectroscopy (EDS) analysis is possible with this instrument, using the Inca 250 SSD XMax20 detector. This technique enables the chemical analysis of the sample via Energy-Dispersive X-Ray Analysis (EDX), where chemical maps and point analyses can be performed, getting to examine the chemical composition of a certain area of study in the sample. The crystal structural properties of the products have been characterized by XRD with the Malvern PANalytical X’pert Pro Materials Powder Diffractometer (MPD), which analyses at room temperature. The X-ray source is a ceramic tube emitting Cu Kα radiation, where λ = 1.540 Å. The geometry of the instrument follows the reflection Bragg-Brentano configuration, in which the sample is kept horizontal, and the system is θ-θ, with a vertical goniometer. The Malvern PANalytical X’pert Pro Materials Research Diffractometer (MRD) has also been used at room temperature. The X-ray source is the same as in the MPD, and the configuration of the device consists of a ω-2θ system with a horizontal goniometer, following the four-circle geometry. Fourier transformed infrared (FTIR) measurements were performed by using PMA50 Tensor 27. Ultraviolet photoelectron spectroscopy (UPS) and X-ray photoelectron spectroscopy (XPS) were performed using a SPECS Phoibos 150 hemispherical energy analyzer. The spectra were calibrated to the Carbon 1s orbital at 284.8 eV, and Al Kα X-ray source for XPS and He source (21.2 eV) for UPS, with a beam width of 100 μm, equipped with auto-neutralization, provided a total energy resolution of 2.9 meV. The contact angle of the films was evaluated using a Drop Shape Analyzer (KRÜSS DSA25S). A precise volume of pure water, 0.937 μL (±0.186 μL), was utilized for the measurements. The test was conducted under controlled conditions, with an average temperature of approximately 20 °C in ambient air.

**Trap density of states (tDOS) measurements by thermal admittance spectroscopy (TAS)**. Temperature-dependent capacitance measurements were carried out in a temperature-controlled sample holder^[4]^. Capacitance-frequency measurements were carried out in a frequency range of 20 Hz to 2 MHz in logarithmic steps using an Agilent E4980A LCR meter. The capacitance-voltage curve was obtained by measuring the capacitance as the applied D.C. bias voltage was scanned from -0.5 to 1.2 V. Based on the capacitance spectra measured at different temperatures, the trap density (*N*_T_) distribution in energy (*E*_ω_) was calculated with the following relations:

$N_{T}(E_{\omega})=-\frac{{´V}_{bi}}{qW}\frac{dC}{d\omega}\frac{\omega}{k_{B}T}$ (1)

$E_{\omega}=k_{B}Tln(\frac{\omega_{0}}{\omega})=k_{B}Tln\left( \frac{2\pi v_{0}T^{2}}{\omega} \right)$ (2)

where *V*_bi_ is the built-in potential and *W* is the depletion width (*V*_bi_ and *W* are derived from capacitance–voltage measurements); *C* is the capacitance measured at an angular frequency *ω* and temperature *T*; *k*_B_ is Boltzmann’s constant and *ω*_0_ is the attempt-to-escape frequency at temperature *T*; and *ν*_0_ is a temperature-independent constant, which can be obtained by fitting the relation of characteristic frequency with different T based on Equation 2.

**Indoor stability measurements** by means of maximum power point tracking (MPPT) were performed using a Litos Lite (Fluxim AG) with constant N_2_ gas flow and connected to a cooling system at fixed temperature of 20 ^o^C under simulated 1-sun using calibrated LED Wavelabs package.

**TEM/ EELS**

High-resolution transmission electron microscopy (HRTEM) and scanning transmission electron microscopy (STEM)–electron energy-loss spectroscopy (EELS) were obtained in an FEI F20 at 200 kV. The HRTEM was acquired with a condenser aperture of 100 μm, no objective aperture, spot size 3, and a BM-UltraScan CCD camera. The STEM–EELS was acquired with a condenser aperture of 70 μm, no objective aperture, nominal camera length 30 mm, spot size 6, and Gatan EF-CCD camera. The EELS supporting STEM images were obtained via an angular dark field detector (DF4).

**Electrical Characterizations of solar cells**

Electrical measurements were carried out using the PAIOS setup purchased from FLUXIM AG equipped with a LED lamp calibrated to 1 sun intensity. All the electrical measurements were done under constant N_2_ gas flow. Electrical impedance spectroscopy (EIS) at dark was performed for bias voltages ranging from 0.2 V to 1 V. with varying frequency from 1Hz to 2 MHz. Moreover, EIS at V_oc_ was measured at light intensities varying from 10% to 100% of the LED’s power. For Mott-Schottky analysis, capacitance was measured 10 kHz with varying voltages from -0.1 to 1.4 volts. Transient photo-current (TPC) measurements were carried out at light-pulse length of 500 μs with settling and follow up times of 500 μs and 200 μs respectively at 10% of light intensity modulation. For open circuit voltage decay (OCVD) measurements light-pulse length of 30 ms along with settling and follow up times of 10 ms and 15 ms were chosen respectively with 10% of light intensity pulses. Finally, the equivalent circuit fittings of Nyquist plots were performed using ZView software. Charge Extraction Linearly increasing voltage (CELIV) was performed with sweeping the voltage from 0 to 1.2, and the ramping rate was changed from 0.005 V/μs to 5 V/μs, with the injection time of 100 μs. Then the mobility of the carriers was calculated based on current injection across the swept voltages.

# Initial assessment of device fabrication using MXene: Me-4PACz as interfacial passivating layer

Although the functionalization of R-MXene seems to be facile and straightforward, their implication in device fabrication which results in the favor of overall power conversion efficiency (PCE) and operational stability of solar cells still needs to be investigated by parameter optimizations. As it is known, Me-4PACz concentration even as an independent HTL self-assembled layer in conventional inverted solar cells should to be optimized, therefore, we functionalized a series of delaminated MXene (R-MXene) with different phosphonic molecules 2PACz, Me-4PACz and H3pp (0.5 mg)[14], with different values for 2PACz (1mg, 2 mg) and Me-4PACz (2 mg, 4.5 mg, 9 mg) and their XRD results are demonstrated in Figure S4 a & b. Then using the prepared functionalized MXenes, devices were fabricated to optimize the best performance of the interfacial passivated device as shown in Figure S4 c&d. they show device parameters including PCE, Fill Factor (FF), open circuit voltage (Voc), and short circuit current density (Jsc) and the tracking of MPP values using ISOS-D-1, respectively. The result indicated that the best performing device is the one passivated by MXene: Me-4PACz (2mg) owing to the improvement mostly in FF value and Voc, which we will refer to this value and type of passivation for the remainder of the study.After this optimization, surface of 3-cation perovskite films were modified using MXene:Me-4PACz deposition. Figure S5 shows SEM surface images of perovskite films with and without MXene:Me-4PACz and Figure S6 a-b shows the EDX-ray spectrum and elemental mapping of MXene:Me-4PACz film covering the surface of perovskite surface. Figure S7 depicts the summary of tracking the device parameters for 3 groups of devices as Control (no passivation as Reference), R-MXene (the device fabricated by deposition of R-MXenes between Halide perovskite and HTL) and MXene: Me-4PACz (the device fabricated by passivation of MXene: Me-4PACz between Halide perovskite and HTL) for 1 month by means of ISOS-D-1(Kept in dark, and N2 atmosphere and JV recording every week). As it is indicated, the best performing device is the MXene: Me-4PACz interfacial passivated device, with PCE about 21.5%, FF about 78%, Voc of 1.16 V and JSC of 23.6 mA.cm^-2^ while the Control champion reached about 20.1% PCE.

In summary, our initial assessment of application of novel MXene nanoneedles functionalized with Me-4PACz demonstrates a significant capability for the enhancement of performance of 3-cation Lead halide perovskite solar cells.

# Supplementary Figures and Tables

**Table S1** XRD parameters of MXene before and after delamination and functionalization in different time.

| **Name** | **Interlayer space (Å)** | **Crystallite size (Å)** | **Nº of layers** |
| --- | --- | --- | --- |
| **MXene:Me-4PACz 2023** | **14.5** | **163** | **11** |
| **MXene:Me-4PACz 2024** | **14.7** | **116.3** | **8** |
| **R-MXene 2024** | **14.1** | **100.8** | **7** |
| **R-MXene 2023** | **14.4** | **153.9** | **10-11** |
| **Bulk MXene** | **9.8** | **166.6** | **17** |

**
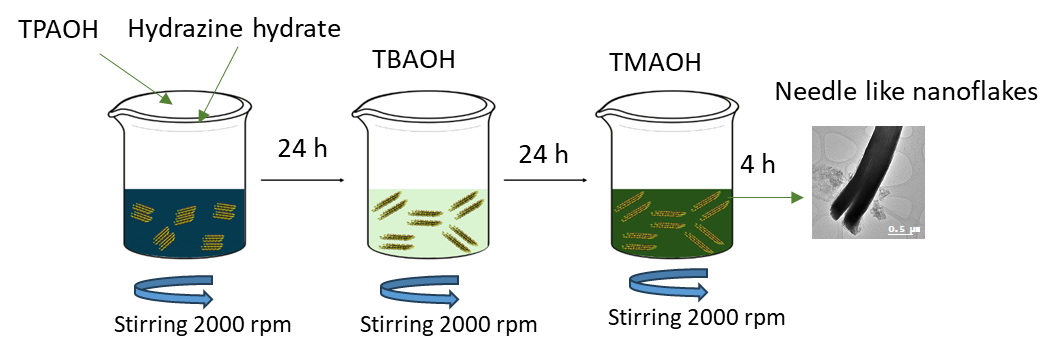
**

**Figure S1.** Bulk MXenes delamination process for the synthesis of Needle-like MXene nanoflakes


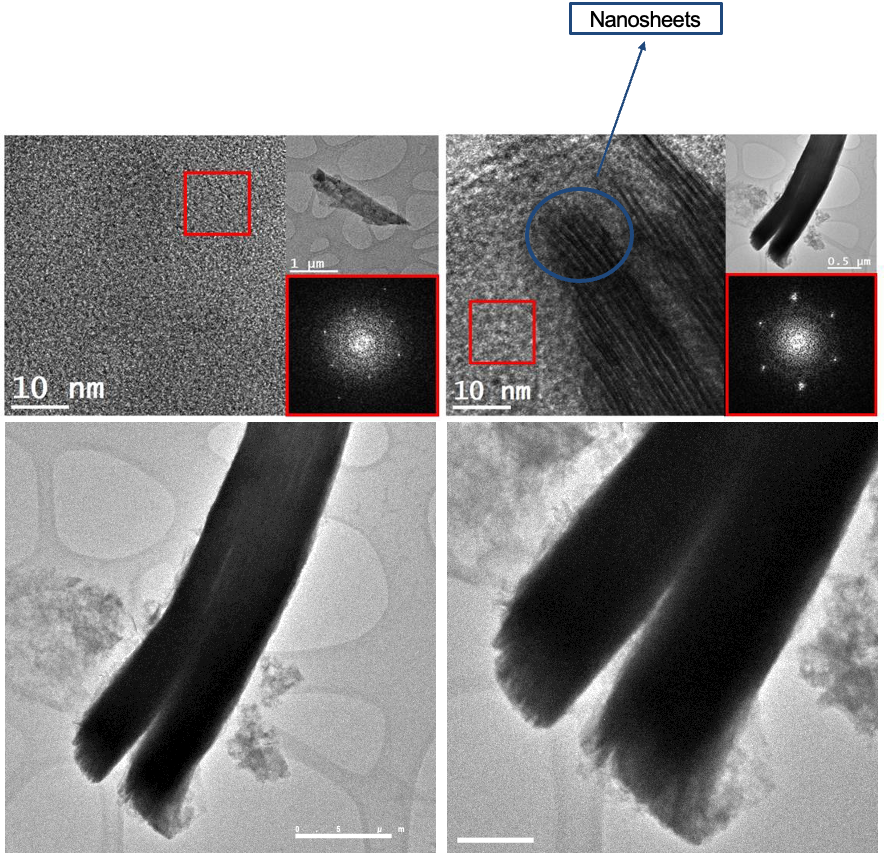


**Figure S2.** HR-TEM images and corresponding power spectrum of the needle-like MXene nano flakes.


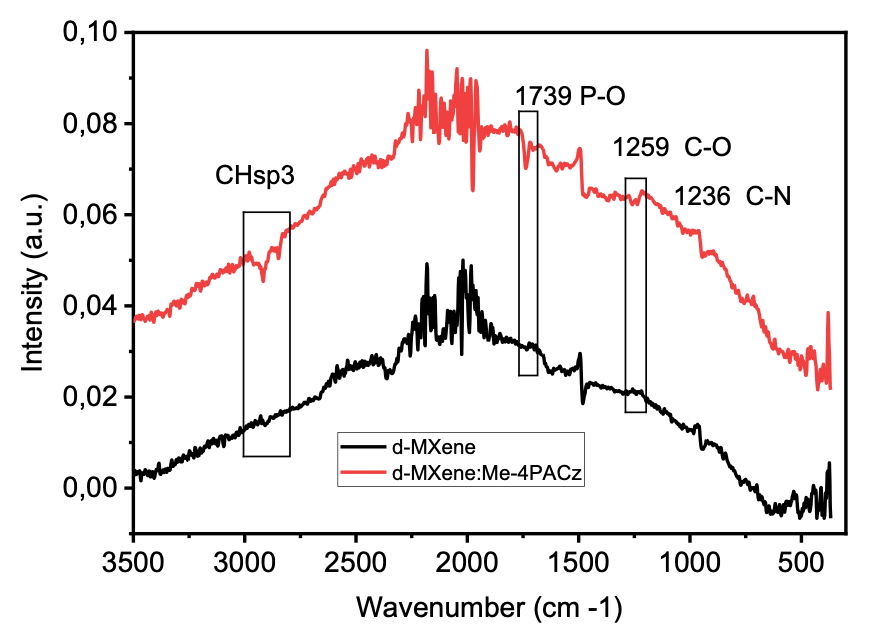


**Figure S3.** FTIR analysis of R-MXene functionalized and delaminated powders


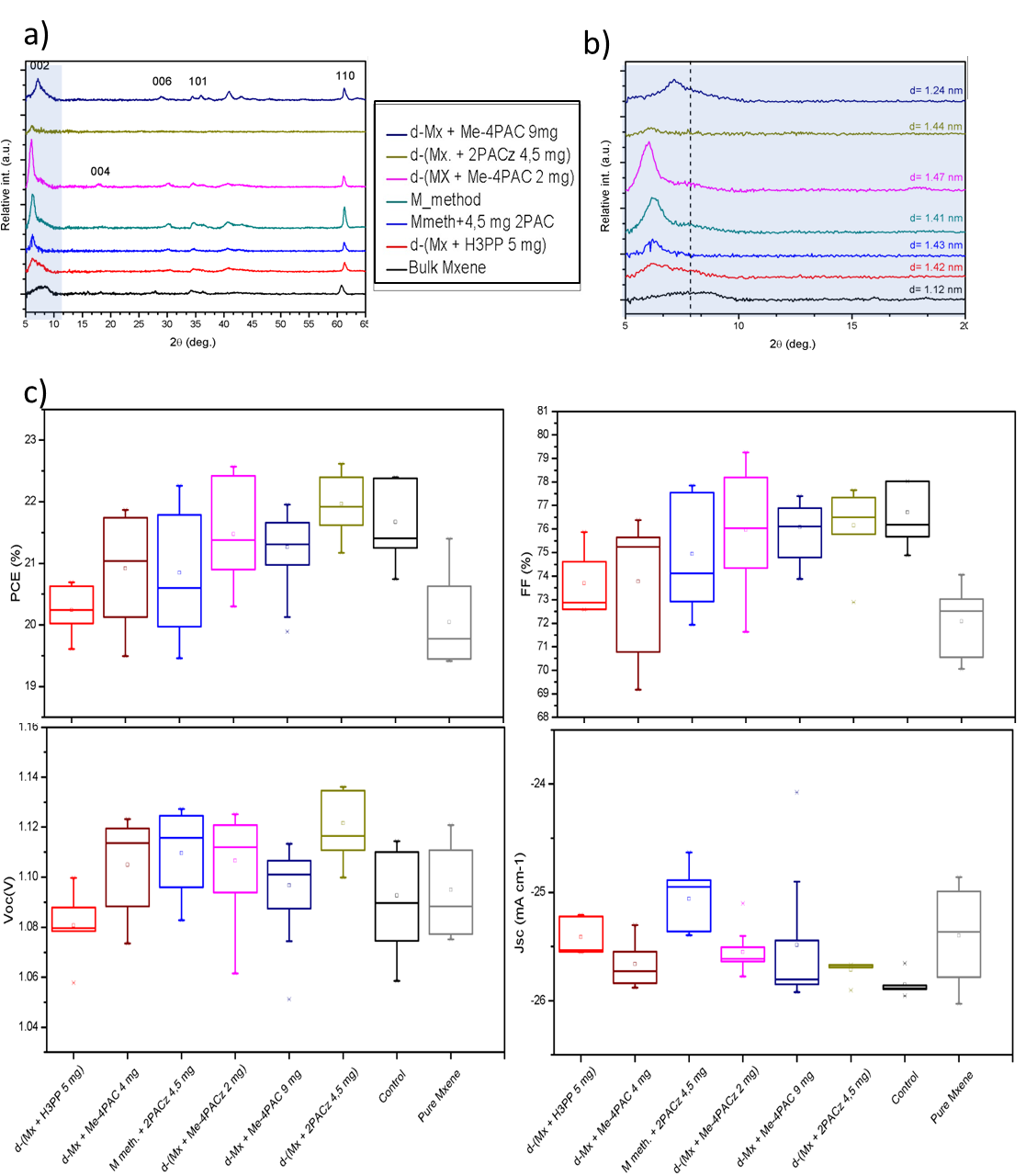


**Figure S4.** Optimization procedure for Bulk MXene, delaminated MXene and functionalized MXene with different chemicals and proportions with H3pp, and two selective hole transfer molecules of Me-4PACz and 2PACz , a&b) overall and zoomed area XRD: c) PCE, Fill factor, Voc and Jsc of devices fabricated with interface passivation using differently functionalizing agents (H3pp, 2-PACz, Me-4PACz) inserted in the interlayers of delaminated R-MXene.


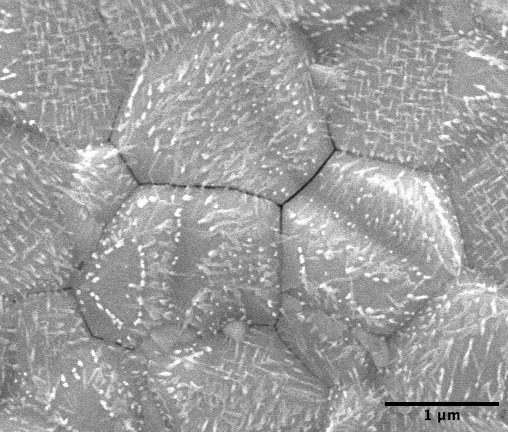

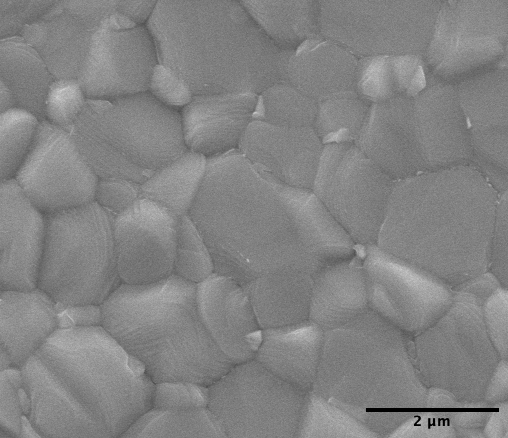


**Modified**

**Control**

**Modified**


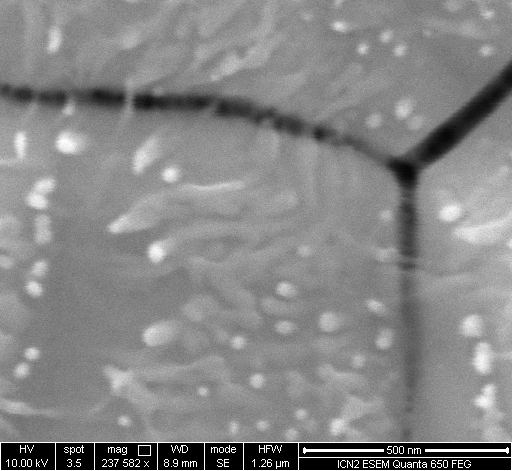


500 nm


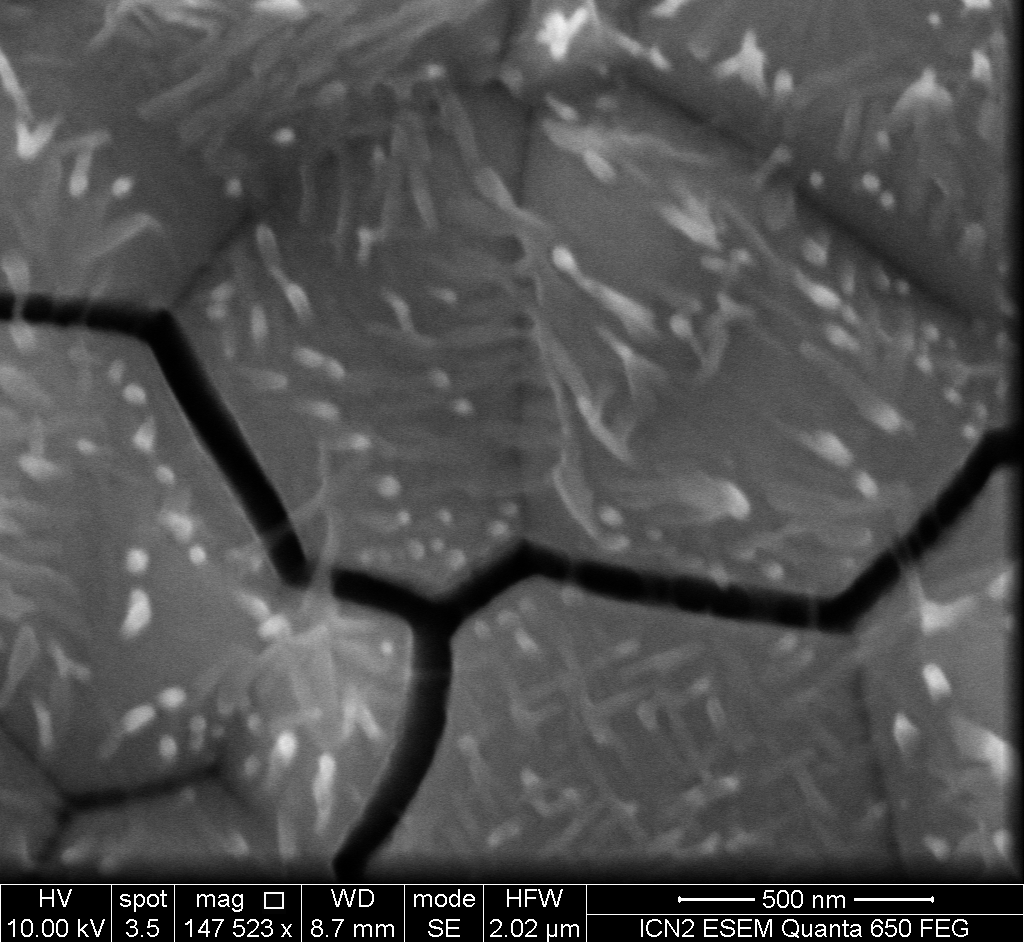


500 nm

**Modified**

**Figure S5**: a) SEM surface images of perovskite films without (control) and with (modified) deposition of MXene:Me-4PACz on it.


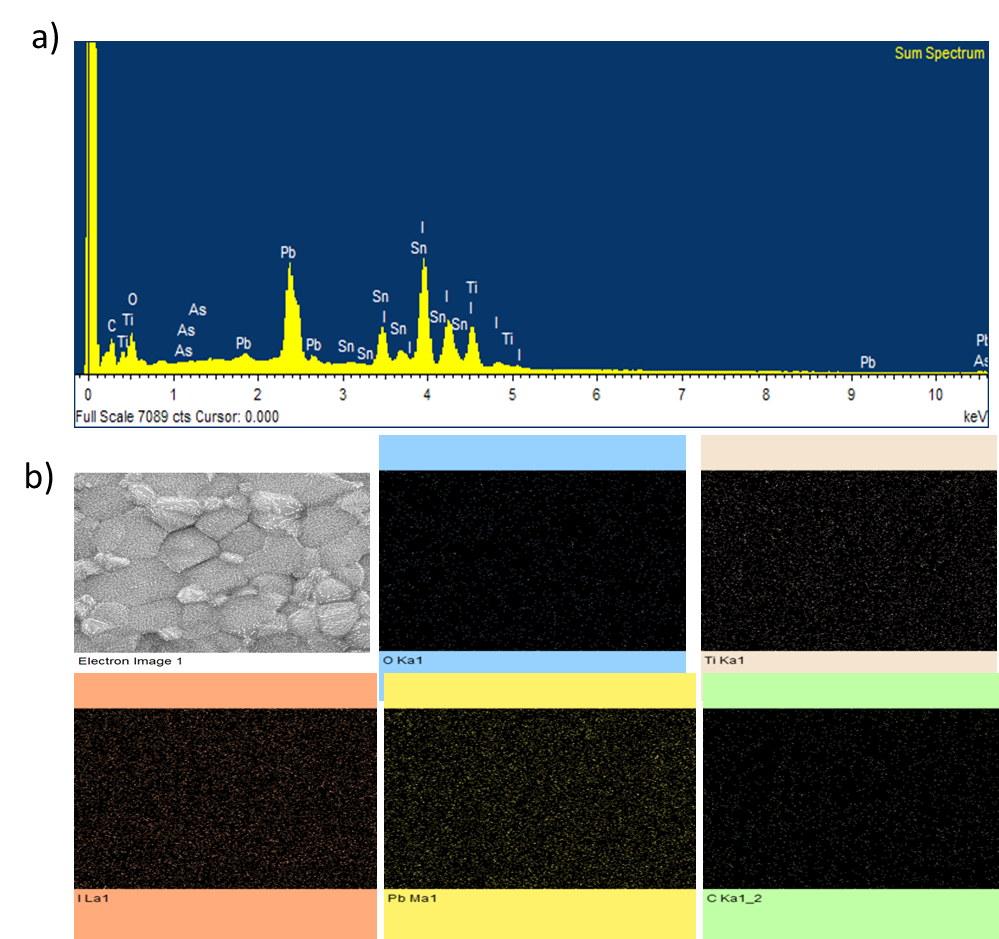


**Figure S6.** a). the Energy Dispersive Spectrum and b) the corresponding elemental mapping for MXene: Me-4PACz modified perovskite film.


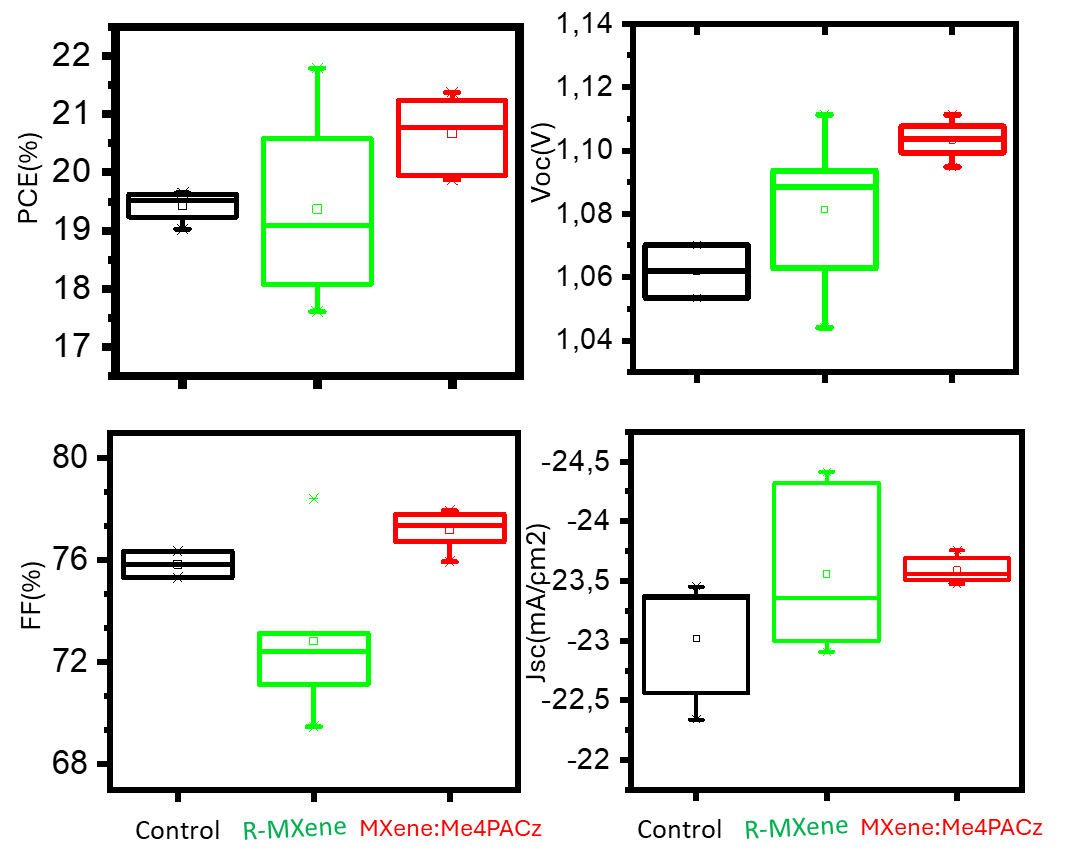


Figure S7: Photovoltaic device parameters of control device, device modified with delaminated MXenes only (R-MXene) and modified with MXene:Me-4PACz (modified).


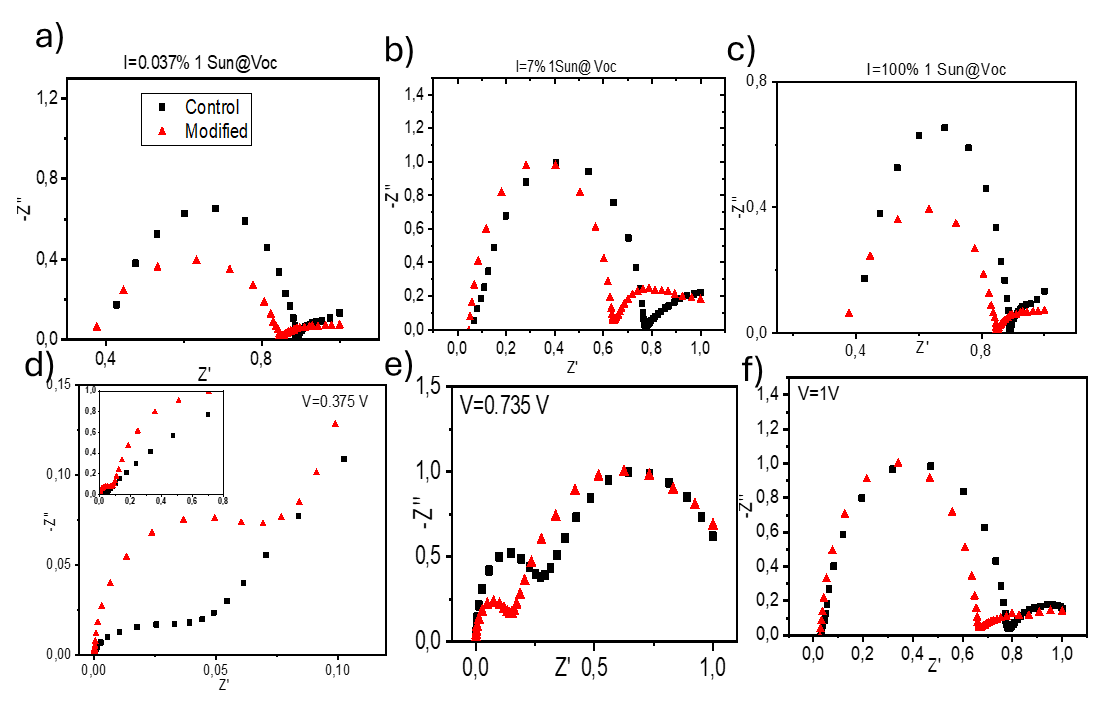


**Figure S8.** Nyquist plots of the champion devices before ISOS-L-1 protocol at a-c) different intensities of simulated 1-sun and, d-e) different voltages in dark condition.


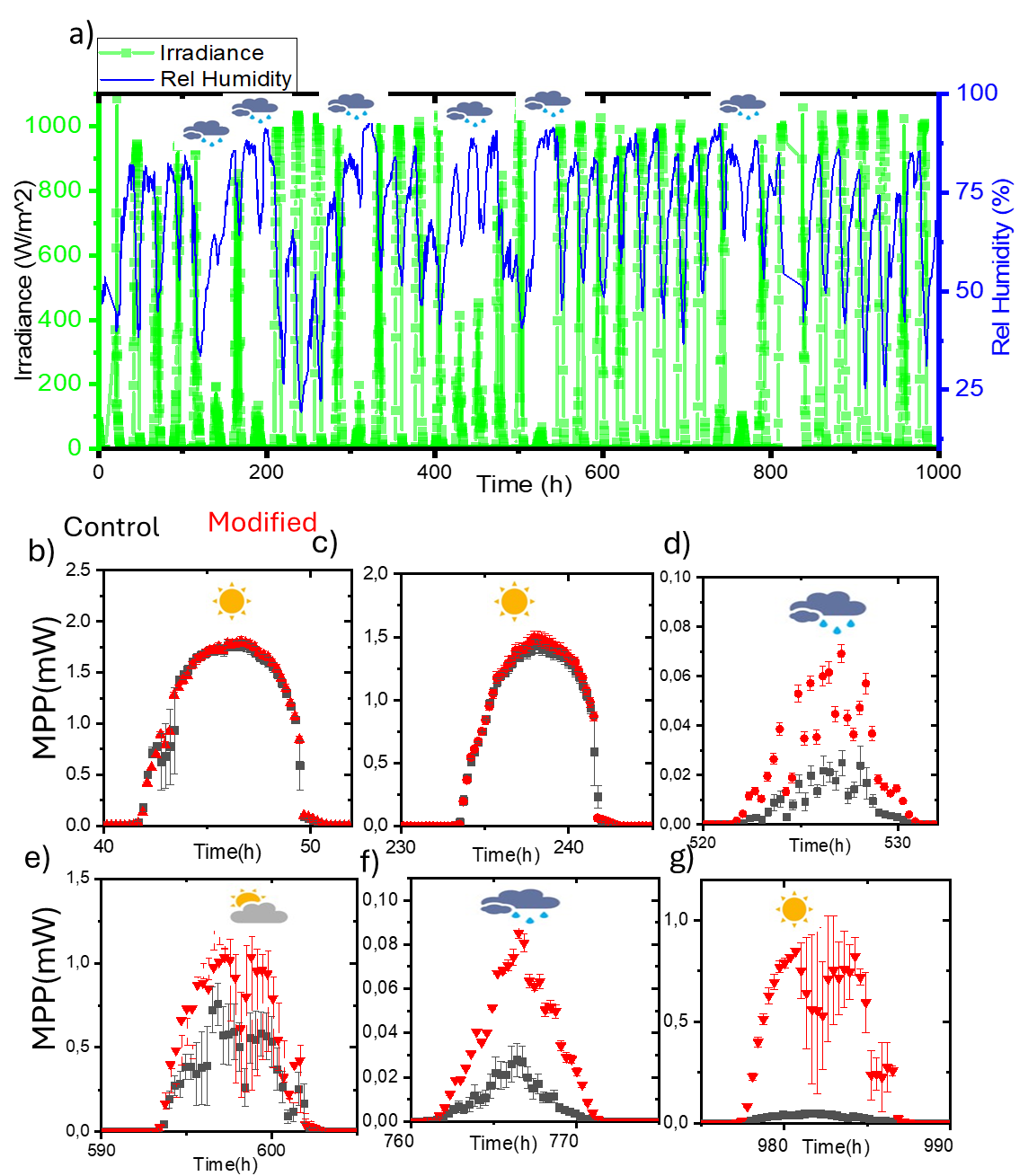


**Figure S9.** a) Humidity and input power tracking of outdoor measurements for ISOS-O-2 stability protocol experiments, b-j) daily evolution of averaged MPP values for the devices in different climate conditions during ISOS-O-2 assessment.


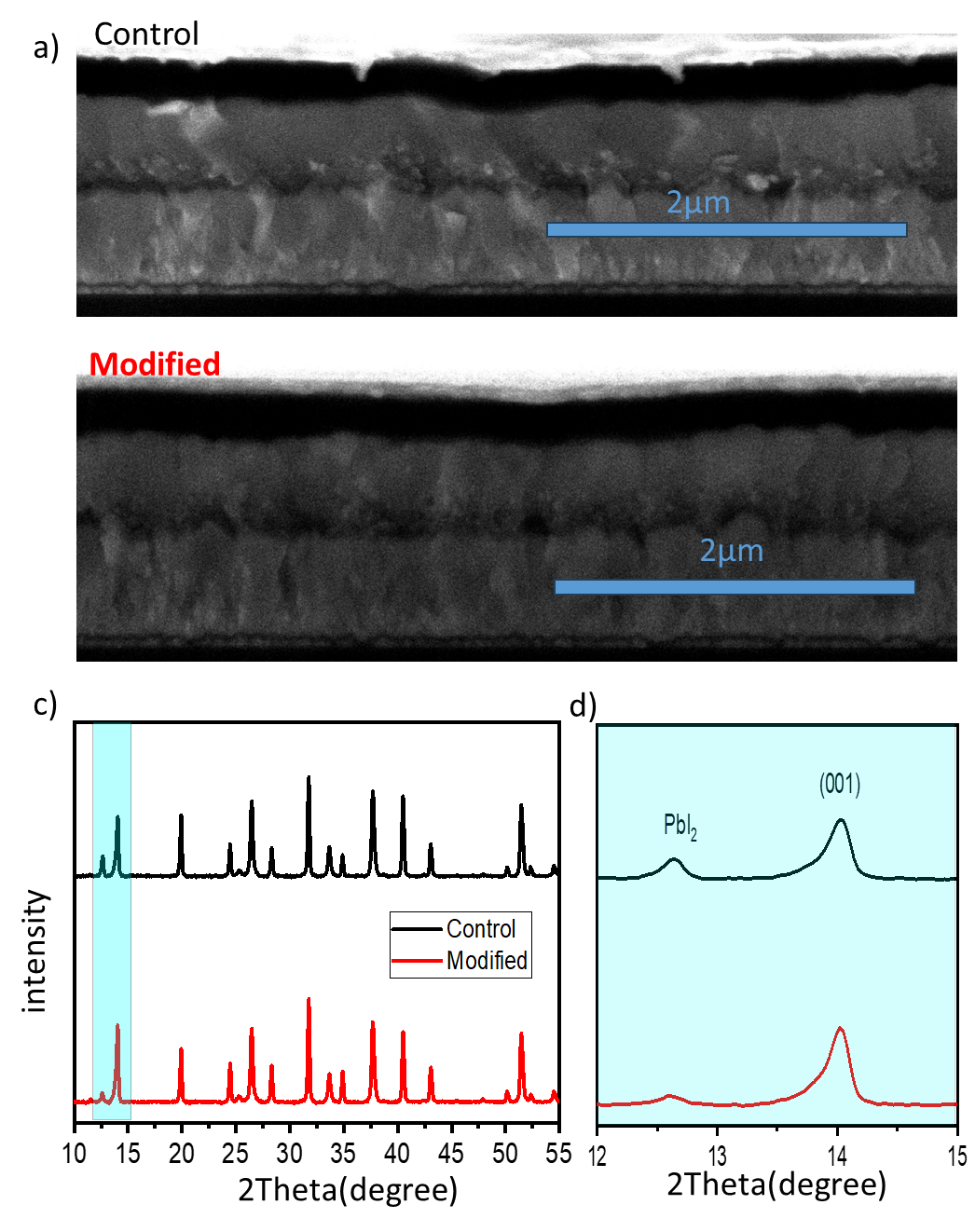


**Figure S10**: a) Large area cross-SEM b) XRD diffractogram of the champion devices after ISOS-L-1 stability assessment.
